# PoTeC: A German naturalistic eye-tracking-while-reading corpus

**DOI:** 10.3758/s13428-024-02536-8

**Published:** 2025-06-30

**Authors:** Deborah N. Jakobi, Thomas Kern, David R. Reich, Patrick Haller, Lena A. Jäger

**Affiliations:** 1 Department of Computational Linguistics, University of Zurich, Andreasstrasse 15, Zurich, 8050 Switzerland; 2 Department of Computer Science, University of Potsdam, An der Bahn 2, Potsdam, 14476 Germany

**Keywords:** Eye-tracking, Reading, German, Corpus

## Abstract

The **Po**tsdam **Te**xtbook **C**orpus (PoTeC) is a naturalistic eye-tracking-while-reading corpus containing data from 75 participants reading 12 scientific texts. PoTeC is the first naturalistic eye-tracking-while-reading corpus that contains eye-movements from domain experts as well as novices in a within-participant manipulation: It is based on a 2$\times$2$\times$2 fully crossed factorial design, which includes the participants’ level of studies and the participants’ discipline of studies as between-subjects factors and the text domain as a within-subjects factor. The participants’ reading comprehension was assessed by a series of text comprehension questions and their domain knowledge was tested by text-independent background questions for each of the texts. The materials are annotated for a variety of linguistic features at different levels. We envision PoTeC to be used for a wide range of studies including but not limited to analyses of expert and non-expert reading strategies. The corpus and all the accompanying data *at all stages of the preprocessing pipeline* and *all* code used to preprocess the data is made available via GitHub: https://github.com/DiLi-Lab/PoTeC and OSF: https://osf.io/dn5hp/. The data is furthermore integrated into the open-source package pymovements, which can be used in Python and R: https://github.com/aeye-lab/pymovements.

## Introduction

Eye-tracking-while-reading data is widely used in different areas of research including linguistics, cognitive psychology, and computer science. In psycholinguistic reading research, it is considered a gold standard dependent variable for investigating the cognitive processes involved in language comprehension (Rayner, 1998; Rayner & Carroll, 2018). An overwhelming majority of eye-tracking studies in psycholinguistic research are based on *controlled* (also referred to as *planned*) *experiments* with hand-crafted stimuli that constitute minimal pairs. However, as recent research has shown, it is crucial to (also) study language processing in ecologically valid settings using naturalistic, real-world stimuli (Demberg & Keller, 2008, 2019; Nastase, Goldstein, & Hasson, 2020).

The stimulus materials of planned experiments, comprising minimal pairs (and relevant filler items), are specifically designed to test a small number of predefined, typically theoretically motivated, hypotheses in a controlled linguistic environment and thus cover only a small range of linguistic constructions. For example, the hypothesis that one syntactic construction is more difficult to process than another one can be investigated by designing minimal pair stimuli that contain the experimental manipulation and certain words or constituents, where the difference in processing difficulty is expected. This experimental approach is essential for making statements about specific phenomena that are often highly infrequent in naturally occurring text. Studying infrequent, complex phenomena can be theoretically relevant as it often allows for teasing apart competing accounts that in many cases make very similar predictions for frequently occurring, simple constructions. Only such a controlled experimental approach allows for drawing conclusions about the causality of observed effects – although one needs to keep in mind that linguistic manipulations are almost always only quasi-experimental in nature.

Conversely, naturalistic reading corpora, where participants read naturally occurring text (e.g., newspaper articles) rather than minimal pair stimuli, cover a wider range of linguistic constructions. This allows for studying a broad and representative range of phenomena without the need to design new experiments and collect new data. The phenomena can be studied within a single dataset and within-subjects, which is not possible in planned experiments where typically one study is limited to a single phenomenon. The coverage of existing theories can be evaluated on these datasets and the theories can be modified accordingly. Additionally, exploratory data analyses can lead to new theories or inspire new research questions (Hamilton & Huth, 2020). While planned experiments using minimal pair stimuli are essential for closely examining and comparing different theories and testing hypotheses, naturalistic corpora permit observational studies in ecologically valid settings, enhancing generalizability, and inspiring new theories based on exploratory data analyses.

Besides the broad categorization into planned experiments with minimal pair stimuli and naturalistic reading corpora, there are more fine-grained differences within naturalistic eye movement corpora. One crucial dimension for characterizing naturalistic eye movement data revolves around the nature of the stimuli, which spans from entirely naturalistic to partially constructed, where, for instance, specific target words within the stimulus sentences are hand-picked based on their lexical frequency. Seminal work on eye-tracking-while-reading corpora, such as the work by Kliegl, Nuthmann, and Engbert (2006), has adopted this approach by using partially constructed stimuli which enables the inclusion of a broad range of linguistic phenomena that are highly infrequent in natural text (but relevant test cases for linguistic theories) while still utilizing sentences that maintain a higher degree of naturalness than typical planned experiments.

Other naturalistic reading corpora leverage naturally occurring text as stimuli that are not specifically designed for the experiment but are simply being reused from an existing source. Often, these texts span multiple sentences, which allows for analyses that go beyond the sentence-level. In the most stringent cases, alterations of the texts are entirely avoided. However, in many cases, texts need to be edited to, for example, compensate for missing context or exclude figures and tables. Leveraging texts that closely resemble or are identical to real-world reading material enables us to study cognitive processes involved in everyday reading at both sentence and text-level, while, as a natural consequence, specific phenomena (e.g., long-distance dependencies) might occur rarely or never within an entire dataset.

In addition to psycholinguistic hypothesis testing, eye-tracking-while-reading data has become increasingly relevant for other areas of research such as natural language processing (NLP). NLP research has been leveraging eye movements in reading for a wide range of tasks such as sentiment analysis (Long, Lu, Xiang, Li, & Huang, 2017; Mishra, Kanojia, Nagar, Dey, & Bhattacharyya, 2017a), named entity recognition (Hollenstein & Zhang, 2019), part-of-speech tagging (Barrett, Bingel, Keller, & Søgaard, 2016) or generating image captions (Takmaz, Pezzelle, Beinborn, & Fernández, 2020) among other tasks. In recent NLP research, eye-tracking-while-reading data has been used to analyze computational language models, for example by investigating their cognitive plausibility (Beinborn & Hollenstein, 2023; Keller, 2010). Sood, Tannert, Frassinelli, Bulling, and Vu (2020) analyzed attention weights learned by transformer language models and compared those to human attention implicitly encoded in human gaze data. More than that, cognitive signals can be used to improve language models (Hollenstein, Barrett, & Beinborn, 2020). Naturalistic reading data can be used to cognitively enhance and augment language models with human gaze data (Deng, Prasse, Reich, Scheffer, & Jäger, 2023; Prasse, Reich, Makowski, Scheffer, & Jäger, 2023; Yang & Hollenstein, 2023; Deng et al., 2024). For all of these research objectives, it is crucial to have large and diverse amounts of cognitive data available. Many NLP tasks rely on long text passages and thus require cognitive data for entire paragraphs of natural texts rather than just single sentences.

In this work, we present the Potsdam Textbook Corpus (PoTeC), a naturalistic eye-tracking-while-reading corpus containing eye-tracking data of German native speakers reading German textbook passages from two different domains. PoTeC is the first corpus to include the level of expertise of each participant as a within-subjects variable and thus allows for analyzing reading strategies used by experts and non-experts. The naturalistic experimental setting of PoTeC encourages various kinds of analyses that are not restricted to test one specific hypothesis and thus have valuable properties that complement other existing corpora.

### New standard for data publication

In addition to the publication of the eye-tracking data, our aim is to foster transparency and re-usability of the data and facilitate leveraging PoTeC for a variety of different use cases (e.g., psycholinguistic research, NLP research, development of new preprocessing algorithms, eye-movement-based biometrics, and many more). In order to achieve this goal, we are presenting a new standard to publish eye-tracking datasets. Following the FAIR principles (**F**indable, **A**ccessible, **I**nteroperable, **R**eusable), our data is shared in the following way (Wilkinson et al., 2016):

- The data is shared via two channels suitable for the respective data type (large data files and code; see Section “Accessing the data”).
- The eye-tracking data is made available *at all stages* (e.g., raw sample data, reading measures data, etc.; see Table 11).
- All the code that was used for preprocessing and analyzing the data is made publicly available in reproducible formats.
- All data and code is accompanied by extensive documentation that not only makes the process transparent and reproducible, but also makes it maximally easy for users with different backgrounds to reuse the scripts for their own purposes.

In addition to following the general FAIR principles, there are two very specific implementations of these principles: As a unique feature of PoTeC, the eye-tracking fixation data is made available both in the original version as it was collected and in a version where the vertical calibration drift was manually corrected post hoc. The availability of this data not only makes the process transparent but also allows to study and compare the original data with the manually corrected data which enables the development of new tools, including machine-learning-based methods, to automatically detect and/or correct vertical drift. The data is furthermore integrated into the open source Python package pymovements^1^. The package allows for building easy machine learning and psycholinguistic pipelines for the processing of eye movement data and can be used in Python and R, which increases re-usability (Krakowczyk et al., 2023).

## Related work

Reading completely naturalistic text passages consisting of multiple sentences, which are shown at the same time, as the texts are presented in PoTeC, exist for several languages, however, only very few for German (see Table 1). A very recently created corpus is the Multilingual Eye-Movements Corpus (MECO-L1) which is a reading corpus containing data from 13 languages. The German subset contains data from 45 participants reading 12 encyclopedic texts on various topics specifically chosen not to require an academic background (Siegelman et al., 2022). The PopSci Corpus includes 17 participants reading 16 popular science texts from different sources in German (Wolfer et al., 2013). WebQAmGaze is another natural reading dataset which includes a German subset (Ribeiro, Brandl, Søgaard, & Hollenstein, 2023). However, the data was recorded using a webcam which results in substantially lower data quality compared to a high-precision eye tracker typically used in eye-tracking-while-reading experiments, and is therefore limited to a small subset of use cases compared to other reading corpora.

The majority of eye-tracking-while-reading datasets exist for English. For example the Provo Corpus: it includes data collected from 84 participants reading 55 short texts from various sources. It also includes human predictability norms (Luke & Christianson, 2017). The Dundee Corpus is one of the earliest eye-tracking-while-reading corpora and it contains data from ten English and ten French native speakers reading newspaper text passages in their native language (Kennedy, Hill, & Pynte, 2003; Kennedy, Pynte, Murray, & Paul, 2013). Another very large corpus is MECO-L2. It includes 543 non-native speakers reading 12 English texts. The participants have 12 different L1 backgrounds^2^ (Kuperman et al., 2023).

**Table 1:** Naturalistic eye-tracking-while-reading corpora for German text passages

Stimuli	Participants	Additional characteristics
**PoTeC (present study)**		
12 texts from undergraduate textbooks on physics or biology ($\# w$: 1896, $\overline{\# w}$: 158 (16))	75 graduate or undergraduate biology or physics students (native speakers) ($\bar{a}$: 24.2 (4.2))	The corpus is designed to specifically enable comparison of expert and non-expert reading. **Eye-tracker**: EyeLink 1000, **Sampl. Freq.**: 1000 Hz
**MECO-L1** (Siegelman et al., 2022)		
12 Wikipedia-style texts on various topics not requiring an academic background ($\# w$: 2028, $\overline{\# w}$: 169)	45 native speakers ($\bar{a}$: 23.76)	Data is available for 12 other languages (nl, en, el, de, he, it, ru, es, tr, ko, no, et and fi)^a^ for a total of 535 participants reading text in their native language. **Eye-tracker**: EyeLink Portable Duo, 1000 or 1000 Plus, **Sampl. Freq.**: 1000 Hz
**PopSci Corpus** (Wolfer et al., 2013)		
16 popular science texts on natural and applied science ($\# w$: 20,000, $\overline{\# w}$: 1250)	17 participants	**Eye-tracker**: EyeLink 1000, **Sampl. Freq.**: 1000 Hz
**WebQAmGaze** (Ribeiro et al., 2023)		
38 Wikipedia-style texts: 2 long texts ($\# w$: 370, $\overline{\# w}$: 185) and 36 shorter texts ($\# w$: 3010, $\overline{\# w}$: 83.6) with questions and annotated answer spans	19 native speakers	The data can be used to study reading during question answering, and is available in two other languages (en, es)^a^. **Eye-tracker**: Webcam, **Sampl. Freq. (mean)**: 25 Hz

All information in the table is taken from the original papers and not all authors report the same information

Abbr.: $\# w$ = total number of words; $\overline{\# w}$ = mean number of words per stimulus text with standard deviation in parentheses; $\bar{a}$ = mean age of participants with standard deviation in parentheses

^a^ Language abbr. (ISO 639-1): nl: Dutch, en: English, el: Greek, de: German, he: Hebrew, it: Italian, ru: Russian, es: Spanish, tr: Turkish, ko: Korean, no: Norwegian, et: Estonian, and fi: Finnish

Many existing corpora use partially constructed single sentences as stimuli instead of text passages. One such corpus is the Potsdam Sentence Corpus (PSC) which includes 33 young and 32 older adults reading 144 individual German sentences (Kliegl, Grabner, Rolfs, & Engbert, 2004; Kliegl et al., 2006). A Turkish reading corpus using partially constructed sentences is TURead which includes data from 196 participants reading 192 stimulus texts each consisting of one to three sentences (Acartürk, Özkan, Pekçetin, Ormanoğlu, & Kırkıcı, 2023). Single-sentence corpora with completely naturalistic stimuli are the very recent RaCCooNS corpus (Frank & Aumeistere, 2023) that includes data from 37 participants reading 200 narrative sentences, and CELER (Corpus of Eye Movements in L1 and L2 English Reading), which is the largest single-sentence corpus (365 participants) with completely naturalistic stimuli: In total, 156 English sentences from the Wall Street Journal were read by 69 native and 296 non-native speakers with five different native language backgrounds.^3^ Half of the sentences in the stimulus corpus were uniquely read by one participant.

Such naturalistic single-sentence experiments offer the benefit of producing data that is simpler to analyze, as it typically avoids multiple lines or pages per stimulus item. Calibration drift complicates fixation-to-line mapping for multi-line text, stimuli cannot be easily placed in the middle of the screen where calibration quality is best but stretch towards the extremes of the screen that are more difficult to calibrate, and re-calibrations are not possible for each sentence or line but only between pages of text. However, applications of single-sentence datasets are restricted to studying reading patterns at the sentence-level, and often at the line-level (i.e., no line-breaks), which excludes the analyses of many daily-life reading scenarios.

Some datasets are specifically designed for developing methods to (automatically) assess properties of the reader or the text, or for being utilized for NLP use cases such as gaze-augmented language modeling. SB-SAT is such a dataset with 95 participants who were asked to judge the subjective difficulty of four passages taken from practice tests for the Scholastic Assessment Test (SAT) (Ahn, Kelton, Balasubramanian, & Zelinsky, 2020). Appendix A provides comprehensive tables additionally listing different types of eye-tracking-while-reading corpora.

### Differences of PoTeC to existing corpora

In sum, the overwhelming majority of naturalistic eye-tracking-while-reading datasets uses English stimulus items with either native or non-native speakers. Moreover, the stimulus materials of the majority of the datasets (be it English or another language) are single (i.e., isolated) sentences rather than paragraphs or texts. Apart from these differences in language and stimulus length, PoTeC differs from already existing datasets in various ways. First, PoTeC is larger than any other existing dataset for naturalistic reading in German in terms of number of participants (for other languages, a few larger datasets do exist). Second, the stimulus texts of PoTeC are relatively demanding and presumably require more cognitive effort to process than, for example, Wikipedia excerpts as used in previous work. This higher difficulty level of the texts is presumably reflected in more complex eye movement patterns. Third, the readers’ domain expertise about the topic presented in the stimulus texts is experimentally controlled and assessed by asking specific background questions on the topics covered in the texts that are not directly answered in the texts, and thus cannot be derived from the read text.

## Methods

**Fig. 1:**
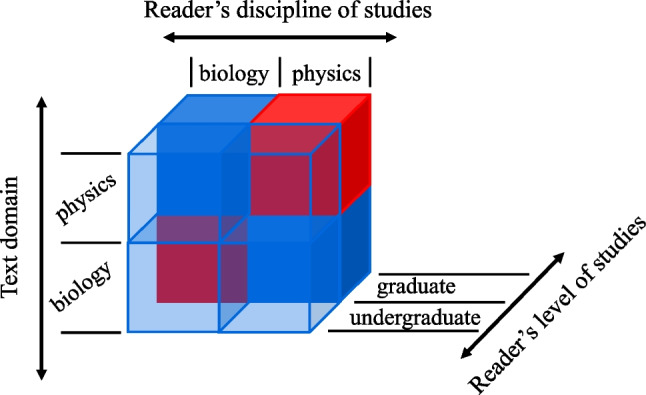
The 2$\times$2$\times$2 fully crossed factorial study design of PoTeC. The *red cubes* denote *expert reading*, that is, a reader having the **level of studies** *graduate* and is reading a text whose **text domain** is equal to the **reader discipline**

PoTeC is an eye-tracking-while-reading dataset using stimulus materials adapted from German university-level textbooks on either physics or biology (see Section “Materials”). The data collection follows a 2$\times$2$\times$2 fully crossed factorial design. The three (quasi-experimental) factors are: 1) The *reader discipline* with the levels *physics* and *biology*, which is manipulated between-subjects and within-items: The selection criteria specify that each participant studies exactly one of the two disciplines; and each item (i.e., each text from one of the domains) is read by the participants from both disciplines. 2) The *text domain* (i.e., the domain of the stimulus texts) with the levels *physics* and *biology* which is manipulated within-subjects and between-items: all readers read both the *physics* and the *biology* texts, and each text belongs unambiguously to exactly one domain. 3) The reader’s *level of studies* with levels *graduate* and *undergraduate* which is manipulated between-subjects and within-items: each reader belongs to exactly one of the groups and each item is read by each of the groups. The level of studies is defined by the semester or program the students are enrolled in at the time of the experiment: *undergraduate* is defined as *first semester BSc*, while *graduate* is defined as being enrolled in an *MSc* or *PhD* program of the respective discipline of studies. See Fig. 1 for an overview of the study design.

In addition to the eye-tracking data, the reader’s text comprehension and text-independent background knowledge were assessed via comprehension and background questions (see Section “Comprehension & background questions”), demographic information was collected from each of the participants (see Section “Demographic questionnaire”), and comprehensive linguistic annotations of the stimulus texts are provided (see Section “Materials”). The eye-tracking data is pre-processed to obtain fixations and reading measures (among other data formats) as described in more detail in Section “Data preprocessing”.

### Materials

The present section provides an overview of the materials used and how the materials were annotated. Table 2 summarizes the different types of annotations and the tools that were used for the different steps of the stimulus annotation process.

#### Stimulus texts

**Fig. 2:**
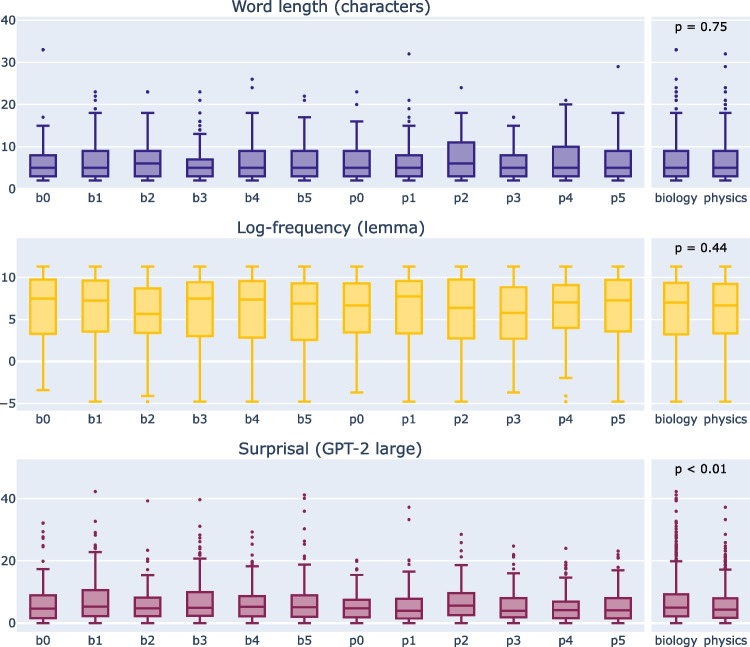
Domain-specific and text-specific summary of word length in characters, the log lemma-frequency, and surprisal (as estimated by GPT-2 large with sentence-context). The *upper/lower frames of the boxes* represent the 25% and 75% quartiles of the data distribution while the *line inside the box* represents the median. The *whiskers* mark the max/min point within all data points that have not been classified as outliers. Outliers are classified as follows: The interquartile range (IQR), which is the length of the box from the upper to the lower frame, is multiplied by 1.5. The box is extended by this length on both sides. All data points that lie outside of this extended range are outliers (represented as dots). The distributions of each characteristic of the two domains have been compared using an unpaired *t*-test assuming non-equal variance

We selected a total of 12 texts from various German university-level physics (six texts) and biology textbooks (six texts) (Ableitner, 2014; Boujard, Anselme, Cullin, & Raguénès-Nicol, 2014; Demtröder, 2010, 2014a, b; Graw, 2015; Townsend, Begon, & Harper, 2003). The original texts were chosen in a way that each text was approximately 150 words long (see Table 3 for the exact number of words for each text and other characteristics, and Section “Accessing the data” for how the stimuli can be accessed). If necessary, the text’s content was adjusted to account for the deletion of mathematical formulas, figures, and tables. The resulting texts were self-contained and consisted only of plain text. The texts enable cross-domain comparisons since they are very similar in terms of text features such as lexical frequency (see Fig. 2, Table 3, and Appendix C). Their main difference lies in their content and not in, for example, texts in one domain simply containing less frequent words. We conducted unpaired *t*-tests with non-equal variance for different text characteristics across the domains, which revealed that only average word surprisal is significantly different between domains (see Fig. 2). However, as surprisal is estimated using a pre-trained language model it is possible that the model was e.g., trained on more text containing terms from one domain. Table 3 also shows that biology texts have on average more expert technical terms which aligns with a high mean surprisal for those texts as well.

**Table 2:** Overview of the tools used to annotate the stimulus materials

Annotation	Data description	Tools
Manual annotation	**Word features (manual)**: stored in one file	Manual
	for each text with one row for each word	STTS tag set (Schiller, Teufel, & Stöckert,
	together with all word level tags (see Section “Manual word-level annotation”).	1999)
Corpus-based annotation	**Word features (extracted)**: stored together with the other word-level tags (see Section “Corpus-based word-level annotation”).	dlexDB online interface^a^ dlexDB (Heister et al., 2011). Lemmata have been manually corrected
Language-model-based annotation	**Surprisal**: The estimated surprisal values are directly added to the files containing the other word-level features (see Section “Language-model-based word-level annotation”).	Pre-trained language models Python scripts: surprisal.py and get_surprisal.py
Semi-automatic annotation	**Dependency & constituency trees**: The trees are created and stored in separate files per tree type, each containing all trees for all texts (see Section “Semi-automatically created dependency and constituency trees”).	Python script: add_syntax_trees.py Python tools: spaCy^b^ and benepar (Kitaev et al., 2019; Kitaev & Klein, 2018) using TIGER grammar (Brants et al., 2004; TIGER Project, 2003). All trees have been manually corrected.

^a^ http://www.dlexdb.de/query/kern/typposlem/

^b^https://spacy.io/

**Table 3:** Text characteristics for each text, and averaged over domains and across all texts

Text domain	Text ID	# Words	# Expert tech.	Mean word	Mean log-	Mean surprisal^b^
			terms^a^	length	frequency (lemma)	(GPT-2 large)
Biology	b0	149	12	6.52 (5.12)	6.19 (4.28)	6.34 (6.60)
	b1	162	18	6.47 (4.47)	6.33 (3.93)	7.45 (7.22)
	b2	150	2	6.95 (4.44)	5.45 (3.97)	6.05 (5.43)
	b3	180	24	5.86 (3.86)	5.82 (4.37)	7.36 (7.00)
	b4	158	11	6.29 (4.40)	5.94 (4.32)	6.41 (5.75)
	b5	155	21	6.63 (3.98)	5.68 (4.34)	6.83 (7.19)
**Mean biology**	**−**	**159 (11)**	**15 (8)**	**6.45 (0.37)**	**5.90 (0.32)**	**6.74 (0.57)**
Physics	p0	176	5	6.21 (3.72)	5.95 (4.04)	5.19 (4.25)
	p1	152	13	6.21 (4.56)	6.22 (4.22)	5.29 (5.61)
	p2	126	8	7.01 (4.32)	6.13 (3.96)	6.63 (5.45)
	p3	175	7	6.20 (3.38)	5.69 (3.67)	5.37 (4.87)
	p4	141	12	6.43 (4.37)	6.23 (3.85)	5.34 (4.82)
	p5	170	22	6.35 (4.12)	6.18 (4.28)	5.47 (4.98)
**Mean physics**	**−**	**157 (20)**	**11 (6)**	**6.40 (0.31)**	**6.07 (0.21)**	**5.55 (0.54)**
**Mean overall**	**−**	**158 (16)**	**13 (7)**	**6.43 (0.32)**	**5.98 (0.27)**	**6.14 (0.82)**

Numbers in parentheses denote the standard deviation of the respective mean. Some characteristics are statistically compared in Fig. 2

^a^ Expert technical terms denote terms that are only understood by experts in the domain of the text (see Table 4)

^b^Surprisal is estimated with the sentence as context (see Table 8)

#### Comprehension & background questions

For each text, three text comprehension questions and three background questions were created. The text comprehension questions required a thorough understanding of the text, but did not require any additional background knowledge. The background questions, in contrast, tested the general knowledge of the topic presented in the text and hence required background knowledge. For example, if a biology text was about molecular biology, the background questions would target knowledge in this area and not just biology in general. The background questions could not be answered with knowledge acquired only through the respective text. All questions had been designed by experts in the respective field. For an example of a stimulus text from each domain and the corresponding questions, refer to Appendix B.

#### Manual word-level annotation

Each stimulus text was manually part-of-speech (PoS) tagged by a trained linguist according to the Stuttgart-Tübingen-Tagset (STTS, Schiller, Teufel, & Stöckert, 1999). In addition to the PoS-tag of the word itself, we provide the PoS-tag of *punctuation marks that directly* (i.e., without a white space) *precede* or *follow the word*, as well as hand-crafted tags to indicate whether a word was contained in a constituent that is *in quotes* or *parentheses*. Furthermore, the words were manually tagged for other lexical and orthographic features that arguably affect eye movement behavior in reading, namely whether the word is an *expert* or *general technical term*, with *expert technical term* being a term that is typically only understood by experts in that area (e.g., biology: “homolog”, physics: “phasenrichtig”) and *general technical term* being a term that is generally understood (e.g., biology: “Proteine”, physics: “Kristalle”). Other tags are whether the word *is* (e.g., “DNA”) or *contains an abbreviation* (e.g., “DNA-Fragment”), *contains a non-Latin character or symbol* (e.g., “$\beta$-D-Glucose”), or *contains a hyphen* (e.g., “z-Richtung”). Finally, we added various ordinal or binary tags to encode positional information for each word (ordinal *position of the word in the text* and *in the sentence*, and whether it is the *first* or *last word of a clause* or *sentence*). See Table 4 for the precise definitions of the different hand-crafted word tags.

**Table 4:** Definitions of the manually created lexical and sentence-level features

Feature	Definition	Example
*Lexical and orthographic features*		
expert technical term	The word is a technical term typically only understood by experts in the field	homolog (biology), phasenrichtig (physics)
general technical term	The word is a technical term but is generally understood without specific expertise required	Proteine (biology), Kristalle (physics)
is abbreviation	The *entire* word is an abbreviation	DNA
contains abbreviation	The word contains an abbreviation	DNA-Fragment
contains symbol	Word contains a symbol	$+$Ende; $\beta$-D-Glucose
contains hyphen	Word contains at least one hyphen that is *not* STTS-tagged as TRUNC	DNA-Fragment; z-Richtung; $\beta$-D-Glucose
*Linear position information*		
word index in text	Position of the word within the current text, irrespective of sentences coded as integer	
word index in sentence	Position of the word within the current sentence coded as integer	
sentence index	Position of the sentence to which the word belongs within the current text coded as integer	
*Punctuation*		
STTS punctuation before	STTS-tag of the punctuation that precedes the word if applicable (Schiller et al., 1999)	
STTS punctuation after	STTS-tag of the punctuation that follows the word if applicable (Schiller et al., 1999)	
quote	Word is (part of an expression that is) in quotes	“Gleisen”
parentheses	Word is (part of an expression that is) in parentheses	(... dielektrische ... )
*Syntactic features*		
clause begin/end	Marks the first/last word of a new clause	
sentence begin/end	Marks the first/last word of a new sentence	
dependency & constituency trees	Dependency & constituency trees for each sentence created semi-automatically	

#### Corpus-based word-level annotation

Moreover, for each word, several word length measures, lexical frequency measures, and lexical neighborhood measures commonly used in reading research were extracted from the lexical database dlexDB (Heister et al., 2011), which is based on the reference corpus underlying the Digital Dictionary of the German Language (DWDS, Das Digitale Wörterbuch der deutschen Sprache, 2016). All extracted values were manually corrected by a linguistic expert labeler. In particular, the type-to-lemma mapping was disambiguated and incorrect database entries (e.g., incorrect lemmatization) were corrected. If the lemma has been manually added, the lemma frequency was coded as a missing value. Overviews of all features extracted from dLexDB are provided in Tables 5, 6, and 7.

**Table 5:** Definition of the corpus-based features extracted from dlexDB: Linguistic representations & word length features

Feature	Definition
Type	Orthographic representation of a word as found in the stimulus text (case sensitive)
Lemma	Headword, i.e., an uninflected form that may or may not occur in the stimulus corpus itself
Syllables	Syllables of which the word consists
Type length char.	Number of characters of a type
Type length syll.	Number of syllables of a type
Lemma length	Number of characters of the lemma

**Table 6:** Definition of the corpus-based features extracted from dlexDB: Frequency measures

Feature	Definition
Annotated type frequency	Number of occurrences of a unique combination of a type, its STTS tag and its lemma in the corpus (per mio tokens)
Type frequency	Number of occurrences of a type in the corpus (per mio tokens)
Lemma frequency	Total number of occurrences of types associated with this lemma in the corpus (per mio tokens)
Document frequency	The number of documents with at least one occurrence of this type (per 10.000 documents)
Sentence frequency	Number of sentences with at least one occurrence of this type (per 100.000 sentences)
Cumulative syllable corpus frequency	Cumulative frequency of the individual syllables of the word in the corpus (per mio tokens)
Cumulative syllable lexicon frequency	Cumulative frequency of the individual syllables of the word as listed in the lexicon (per mio types)
Cumulative character corpus frequency	Cumulative corpus frequency of all characters contained in this type (per mio tokens)
Cumulative character lexicon frequency	Cumulative lexicon frequency of all characters contained in this type (per mio types)
Cumulative character bigram corpus frequency	Cumulative corpus frequency of all character bigrams contained in this type (per mio tokens)
Cumulative character bigram lexicon frequency	Cumulative lexicon frequency of all character bigrams contained in this type (per mio types)
Cumulative character trigram corpus frequency	Cumulative corpus frequency of all character trigrams contained in this type (per mio tokens)
Cumulative character trigram lexicon frequency	Cumulative lexicon frequency of all character trigrams contained in this type (per mio types)
Initial letter frequency	Cumulative frequency of all types sharing the same initial letter (per mio tokens)
Initial bigram frequency	Cumulative frequency of all types sharing the same initial character bigram (per mio tokens)
Initial trigram frequency	Cumulative frequency of all types sharing the same initial character trigram (per mio tokens)
Average conditional probability in bigrams	Conditional probability of the respective word being the second component in word bigrams, given the occurrence of its first component, averaged across all possible bigrams with that word as second component (computed on the basis of the annotated type information)
Average conditional probability in trigrams	Conditional probability of the respective word being the third component in word trigrams, given the occurrence of its first and second component, averaged across all possible trigrams with that word as third component (computed on the basis of the annotated type information)
Familiarity	Cumulative frequency of all types of the same length sharing the same initial trigram
Regularity	The number of types of the same length sharing the same initial trigram

#### Language-model-based word-level annotation

In addition to the manual tagging and the dlexDB tags, all words in the texts were annotated with surprisal. For a given word $w_i$, its surprisal $s_i$ with the given context $\textbf{w}_{<i}$ is defined as $s_i = -\log p(w_i \mid \textbf{w}_{< i})$. As has been shown by previous research, surprisal values and their predictive power differ depending on the language model that was used for their estimation (Goodkind & Bicknell, 2018; E. G. Wilcox, Gauthier, Hu, Qian, & Levy, 2020; E. Wilcox, Meister, Cotterell, & Pimentel, 2023). Therefore, the surprisal values were estimated by three models that differed in architecture and size. Two models had been trained in an auto-regressive manner which takes only the left-hand side context of a word into account to predict the next word, which mimics the incremental nature of human language processing. The first is GerPT2 (Minixhofer, 2020), a language model that was trained on German data but initialized from the English GPT-2 model (Radford et al., 2019). Both GerPT2 Large^4^ (876M parameters) and Base^5^ (176M parameters) were used. The second model is LeoLM, a German language model built on Llama 2 (Touvron et al., 2023) and is fine-tuned on German data. We used the 7b^6^ and 13b^7^ versions of LeoLM. The third model used is a German variant^8^ of BERT (Devlin, Chang, Lee, & Toutanova, 2019) with 109M parameters. BERT is not an auto-regressive model as it takes both the left- and right-hand context into account. In this case, surprisal $s_i$ for one word $w_i$ with the given context $\textbf{w}_{<i}, \textbf{w}_{>i}$ is defined as $s_i = -\log p(w_i \mid \textbf{w}_{< i}, \textbf{w}_{> i})$.

**Table 7:** Definition of the corpus-based features extracted from dlexDB: Neighborhood measures

Feature	Definition
Coltheart neighborhood measures: all measures are based on the definition of **neighbor** of Coltheart, Davelaar, Jonasson, and Besner (1977).	
Neighbors need to be of the same length and differ at one character position from each other. E.g., “Hans” and “Haus” are Coltheart neigbors.	
Levensthein neighborhood measures: all measures are based on the definition of **neighbor** of Levenshtein (1966). Words are neighbors if they	
differ by one change operation (inserting, deleting or exchanging a character). E.g., Brot und rot are Levenshtein neigbors.	
Cumulative frequency of higher frequency neighbors (Coltheart & Levensthein)	Cumulative frequency of all higher frequency orthographic neighbors
Count of higher frequency neighbors (Coltheart & Levensthein)	Number of higher frequency orthographic neighbors
Cumulative frequency of all neighbors (Coltheart & Levensthein)	Cumulative frequency of all orthographic neighbors
Count of all neighbors (Coltheart & Levensthein)	Number of orthographic neighbors

Note: all four measures are computed once for the Coltheart and once for the Levensthein definition resulting in a total of eight measures

In order to obtain the surprisal values, all texts were sub-word tokenized for the respective model, and the tokenized input sequence was processed by the model to obtain the log-probabilities of the individual sub-word tokens which were then added up to get the surprisal for each word. All surprisal values were estimated once with the respective (left-hand) sentence as context and once with the entire (left-hand) text as context as the participants had also seen the entire text on one page during reading (Table 8).

#### Semi-automatically created dependency and constituency trees

To facilitate future analysis of the data, dependency and constituency trees were added to all sentences. In order to create the dependency trees, the Python library spaCy^9^ was used. The tool to create the dependency trees was trained on the TIGER corpus and it therefore uses the TIGER annotation scheme (Brants et al., 2004; TIGER Project, 2003). The tool automatically parses and annotates each word with its respective dependencies. The dependency trees were manually corrected by a German linguistic expert. The constituency trees were created using the open-source tool benepar (Kitaev & Klein, 2018; Kitaev, Cao, & Klein, 2019). The tool receives each text separately as input, splits it into sentences, tokenizes the sentences, annotates the words with PoS-tags and groups the resulting annotated words into sentence constituents and annotates them accordingly. As the entire pipeline relies on pre-trained models for the different tasks, it might result in different PoS-tag annotation compared to our manual tags. The resulting constituency trees were consequently manually corrected such that the PoS-tags correspond with our manual tags and to account for any other errors.

### Participants

75 native speakers of German with normal or corrected-to-normal vision who studied at the University of Potsdam (Germany) participated in the experiment.^10^ They were either students of *biology* or of *physics* in either their first semester of the BSc program (*undergraduate*) or graduate students currently attending an MSc or PhD program (*graduate*). In total, there were 12 undergraduate physics students, 20 graduate physics students, 16 undergraduate biology students, and 27 graduate biology students.

Participants were requested to not have consumed any alcohol on the day of the experiment and come to the experiment well rested. Participants received a compensation of 20 EUR. A short overview of the participants’ mean age and a selection of other characteristics are presented in Table 9.

### Experiment procedure and technical set-up

The data collection was carried out in accordance with the Helsinki Declaration (World Medical Association, 2013). Informed consent was obtained from all participants prior to starting the experiment.

**Table 8:** Surprisal estimates by different types of language models are included as features for each word

Model type	Size
**Auto-regressive**	
GerPT2 base (GPT-based)	176M parameters
GerPT2 large (GPT-based)	876M parameters
LeoLM 7b (Llama-2-based)	7B parameters
LeoLM 13b (Llama-2-based)	13B parameters
**Non-auto-regressive**	
BERT	190M parameters

For all model types, surprisal has been estimated once with the sentence and once with the entire text as context. As the surprisal is estimated on sub-word-token-level, the resulting surprisal values have been added up to obtain the surprisal for one word

**Table 9:** Overview of the participants’ mean age and a selection of other characteristics

Reader discipline	Level of studies	Number of participants	Mean age	Mean hours of sleep	Glasses
Biology	undergrad.	16	21.5 (3.2)	7.3 (0.9)	no: 14, yes: 1, N/A: 1
Biology	graduate	27	26.2 (4.1)	7.3 (1.0)	no: 17, yes: 10
Physics	undergrad.	12	20.5 (3.3)	6.6 (2.3)	no: 8, yes: 4
Physics	graduate	20	25.7 (2.8)	7.4 (1.2)	no: 15, yes: 5
**Overall**		**75**	**24.2 (4.2)**	**7.2 (1.3)**	**no: 54, yes: 20, N/A: 1**

The standard deviation for mean values is added in parentheses

Participants were instructed about the procedure of the experiment in written form and clarified their questions with the experimenter orally. The participants were instructed to read the texts silently. Very importantly, it was pointed out that they should read each text such that they understand the content thoroughly. They were informed that they will have to answer comprehension questions some of which are hard to answer and even require knowledge that might go beyond the knowledge presented in the text. In that case, they should pick the answer that seems most plausible. The full instructions, which also contain specifics about how they should be seated etc., can be found in Appendix E and in the repository. The experiment started with the recording of the eye movements (see Section “Eye-tracking-while-reading”) which was followed by a short demographic questionnaire (see Section “Demographic questionnaire”). The total duration of the experiment including instructions, camera setup, breaks, calibrations, and questionnaire was approximately 90 minutes.

#### Eye-tracking-while-reading

Participants’ eye movements (right eye monocular tracking) were recorded at a sampling rate of 1000 Hz using an EyeLink 1000 eye tracker manufactured by SR Research with a desktop-mounted camera system with a 35-mm lens. A Cedrus button box was used as a response pad. The experimental presentation and the communication between the presentation computer and the eye tracker was implemented using the Experiment Builder software provided by SR Research.

The participant was seated at a height-adjustable table to ensure a constant eye-to-screen distance across participants. The participant’s head was stabilized using a chin- and forehead rest, which should increase the quality of the recorded data. The texts were presented on a 22-inch monitor with a resolution of 1680$\times$1050 pixels and a screen size of 47.5$\times$30 cm. The eye-to-screen distance measured 61 cm and the eye-to-camera distance was 65 cm. Both distance measures were measured as specified in the EyeLink Installation Guide (SR Research Ltd, 2010, p. 15, p. 70).

The texts were presented in a mono-spaced white font (Courier, font size 18) on a black background. The reason for choosing a black background was the rather long duration of the experiment. A bright background color would strain the participants’ eyes and potentially lead to wet eyes, which has a negative impact on calibration accuracy.

**Table 10:** Statistics over the number of calibrations and validations performed in the experiment including the validation scores

Type	Validation score (avg)				Validation score (max)				# per session			
	mean	std	max	min	mean	std	max	min	mean	std	max	min
cal									14.418	4.73	27	2
val	0.421	0.253	2.37	0.188	0.84	0.567	5.045	0.422	8.899	3.07	15	2

Note: The validation scores (avg and max) are both averages over all the validations performed in one session while the original scores are either the maximum or the average across all target points of one validation. SR research recommends an average validation score below 0.5^a^. That is for *each validation performed in one session* the average score across all target points should be below 0.5. See Appendix D for a session overview

^a^ Specified in an SR Research blog post (needs an account to access it): https://www.sr-research.com/support/showthread.php?tid=244

After the set-up and initial calibration (nine-point calibration) of the camera and the validation, the participant first read one practice text followed by six practice questions to get familiar with the experimental procedure. The twelve experimental texts were presented in randomized order (separate randomization for each participant). Each experimental trial began with the presentation of the header of the following text on an individual screen. The participant had to press a button to continue to the text which was shown on a new screen. Each text fit onto a single screen. There were no restrictions regarding the time spent on reading each text. After having finished reading the text, the participant had to look at a green sticker that was placed on the bottom right corner of the monitor and at the same time press a button to continue to the questions. This procedure helps to avoid random fixations on the text after the participant has finished reading as they are fixating a specified target away from the areas of interest. Each question was presented on a separate screen together with four answer options of which the participant had to select one by pressing the respective button on the response pad. It was not possible to go back to the text or previous questions, nor was it possible to undo an answer. The order of answer options was randomly shuffled for each participant. The three text comprehension questions always preceded the three background questions in order to minimize memory effects on the response accuracy; the order of the three questions within each type was randomized for each participant. Participants were informed that some of the questions required background knowledge, however they were not informed which ones.

If necessary, re-calibrations were performed before the beginning of a new trial followed by another validation. For all of the participants, re-calibrations were performed throughout the experiment (see Table 10 for an overview on the calibrations and validations performed). Appendix D presents a table where all the average validation scores for each session are listed together with the number of validations and calibrations performed in that session. Participants were allowed to take a break before the beginning of a new trial.

#### Demographic questionnaire

After the eye-tracking experiment was concluded, participants had to fill in a short demographic questionnaire. The following information was collected: the field of studies (including area of specialization if applicable) and the current semester of studies to verify the level of studies, the grade of the German university entrance diploma, gender, age, handedness, whether the participant was wearing (soft or hard) contact lenses or glasses, hours of sleep the night before the experiment, alcohol consumption within 24 hours before the experiment, whether or not the participant had grown up bilingually, and the state (“Bundesland”) where the German language was acquired.

### Data preprocessing

The eye-tracking data for each participant and text were pre-processed, and are made available in different formats. Whenever the tools and scripts are created by ourselves and not protected by a license or copyright, the tools used to complete the different steps are made publicly available. Table 11 provides an overview of the different pre-processing steps and the tools used.

#### Pre-processing of raw data

The data files originally written by the eye-tracking device are non-human readable edf files which were directly converted to a human-readable asc format using the SR Research edf2asc tool. The asc files contain the data for one session including metadata and need to be parsed to extract the relevant samples for each trial and store it in a separate tsv file which then contains one sample per line for one trial. One sample consists of the *x* and *y* coordinates for the tracked eye and the timestamp.

#### Computation of fixations

Fixations and saccades were computed from the pre-processed raw data using the EyeLink Data Viewer software provided by SR Research with the default parameter settings (SR Research Ltd., 2011). Subsequently, each fixation was mapped to the character index in the text that was fixated and annotated with the line index and the character index in that line. Fixations on the white space between two words were mapped to the closest character.

**Table 11:** Data processing pipeline including the tools that were used

Pre-processing Step	Data description	Tools
**Pre-processing of eye movement data**		
*Collect*: raw data	**Raw data (as-is)**: Raw data including metadata *for each session* in one file. At experiment time, non-human-readable edf files are written that are directly converted to human-readable asc files.	EyeLink 1000, Experiment Builder software (SR Research), SR Research edf2asc
*Pre-process*: raw data	**Raw data (pre-processed)**: Raw data as collected is pre-processed to only contain the relevant samples (right eye *x* and *y* coordinates, pupil diameter, timestamp) *for each trial*. The files contain one data sample per line (tsv format).	asc_to_csv.py, SR Research asc2csv
*Compute*: raw data $\rightarrow$ fixation data	**Uncorrected fixation data**: From the pre-processed raw data, fixations are computed. Contains the character-level area of interest for each fixation and fixation and saccade features.	Eyelink Data Viewer (SR Research Ltd., 2011), default parameter settings
*Correct*: fixation data $\rightarrow$ corrected fixation data	**Fixations**: Manually corrected fixation data. Contains information about whether the fixation was corrected or not and if so, information on the original fixation (see Section “Manual correction of the fixation data”).	Python script: correct_fixations.py and split_fixation_ report.py
**Pre-processing of stimulus material**		
*Compute*: character index $\rightarrow$ word index	**Character to word mapping**: The indices of the character-level areas of interest are mapped to the word indices in the respective text.	Python script: char_index_to_ word_index.py
*Compute*: text $\rightarrow$ word/sentence limits	**Word limits and sentence limits**: Contains information about the first and the last character index (i.e., aois) of each word and each sentence in each text.	Python script: create_word_ aoi_limits.py
**Pre-processing of stimulus material and eye movement data combined**		
*Compute*: corrected fixation data $\rightarrow$ word-level reading measures	**Reading measures**: For each trial, word-level reading measures are computed that are written to a separate file for each reader and text (see Section “Computation of word-level reading measures”).	Python script: compute_reading_ measures.py
*Merge*: corrected fixation data with character and words	**Scanpaths**: Each character-level fixation in all scanpaths was merged with the fixated character and word, and information on trial-level like the text identifier is added (see Section “Summary of the available features”).	Python script: generate_scanpaths.py
*Merge*: reading measures with stimulus corpus data	**Reading measures merged**: Merge reading measures with all features at all levels that have not been included yet (see Table 15 for an overview)	Python script: merge_reading_ measures.py
*Merge*: scanpaths with stimulus corpus data	**Scanpaths merged**: Merge scanpaths with all features at all levels that have not been included yet (see Table 15 for an overview).	Python script: merge_scanpaths.py

**Table 12:** Definition of the word-level eye movement measures

Measure	Abbr.	Definition
**Continuous measures (in ms)**		
first-fixation duration	FFD	duration of the first fixation on a word if this word is fixated in first-pass reading, otherwise 0
first duration	FD	duration of the first fixation on a word (identical to FFD if not skipped in the first-pass)
first-pass reading time	FPRT	sum of the durations of all first-pass fixations on a word (0 if the word was skipped in the first-pass)
single-fixation duration	SFD	duration of the only first-pass fixation on a word, 0 if the word was skipped or more than one fixations occurred in the first-pass (equals FFD in case of a single first-pass fixation)
first-reading time	FRT	sum of the duration of all fixations from first fixating the word (independent if the first fixations occur in first-pass reading) until leaving the word for the first time (equals FPRT in case the word was fixated in the first-pass)
total-fixation time	TFT	sum of all fixations on a word (FPRT$+$RRT)
re-reading time	RRT	sum of the durations of all fixations on a word that do not belong to the first-pass (TFT−FPRT)
inclusive regression-path duration	RPD_inc	sum of all fixation durations starting from the first first-pass fixation on a word until fixating a word to the right of this word (including all regressive fixations on previous words), 0 if the word was not fixated in the first-pass (RPD_exc$+$RBRT)
exclusive regression-path duration	RPD_exc	sum of all fixation durations after initiating a first-pass regression from a word until fixating a word to the right of this word, without counting fixations on the word itself (RPD_inc−RBRT)
right-bounded reading time	RBRT	sum of all fixation durations on a word until a word to the right of this word is fixated (RPD_inc−RPD_exc).
**Binary measures**		
fixation	Fix	1 if the word was fixated, otherwise 0 (FPF or RR)
first-pass fixation	FPF	1 if the word was fixated in the first-pass, otherwise 0
first-pass regression	FPReg	1 if a regression was initiated in the first-pass reading of the word, otherwise 0 (sign(RPD_exc))
re-reading	RR	1 if the word was fixated after the first-pass reading, otherwise 0 (sign(RRT))
**Ordinal measures**		
total fixation count	TFC	number of all fixations on a word
landing position	LP	position of the first saccade on the word expressed by ordinal position of the fixated character
incoming saccade length	SL_in	length of the saccade that leads to first fixation on a word in number of words; positive sign if the saccade is a progressive one, negative sign if it is a regression
outgoing saccade length	SL_out	length of the first saccade that leaves the word in number of words; positive sign if the saccade is a progressive one, negative sign if it is a regression; 0 if the word is never fixated
total count of outgoing regressions	TRC_out	total number of regressive saccades initiated from this word
total count of incoming regressions	TRC_in	total number of regressive saccades landing on this word

#### Manual correction of the fixation data

Visual inspection of the fixation data revealed that in certain fixation sequences, vertical calibration error gradually increased over time. This measurement error was manually corrected by adjusting the fixation-to-character mapping (i.e., re-mapping a fixation to the character in the line above or below the currently mapped character). Horizontal calibration drift could not be corrected as there is no way to infer the magnitude of the measurement error from the data. An exception is fixations on the first or last word of a line. If the fixation was just next to the line and not on the word, it was corrected to be on the first, respectively last word of the line. In any case, horizontal measurement error is less dramatic for the purpose of this study (and reading experiments in general) as fixations are eventually mapped to words, thus a horizontal measurement error will only result in a wrong fixation-to-word mapping if the measurement error is larger than the distance of the real fixation location to the word boundary. In contrast, even small measurement error on the vertical axis can lead to incorrectly mapping the fixation to a word in the line above or below.

**Table 13:** Mean and standard deviation (in parentheses) of a selection of commonly used reading measures **for each text**

Text	SFD	FFD	FPRT	FPReg	RPD_inc	TFT	RRT
b0	116 (132)	160 (133)	261 (434)	0.142 (0.349)	545 (2925)	545 (792)	285 (558)
b1	111 (131)	159 (131)	243 (295)	0.161 (0.368)	664 (4304)	664 (791)	421 (681)
b2	120 (135)	172 (128)	252 (265)	0.161 (0.368)	627 (4305)	627 (728)	375 (626)
b3	117 (138)	164 (136)	249 (308)	0.169 (0.375)	661 (4343)	661 (754)	412 (621)
b4	111 (132)	161 (131)	258 (361)	0.150 (0.357)	592 (3691)	592 (780)	334 (605)
b5	120 (136)	176 (133)	275 (316)	0.162 (0.369)	724 (4374)	724 (740)	449 (606)
p0	126 (140)	170 (135)	234 (238)	0.159 (0.366)	564 (3007)	564 (537)	329 (446)
p1	119 (135)	166 (133)	242 (287)	0.174 (0.379)	632 (3380)	632 (657)	389 (542)
p2	118 (138)	170 (136)	258 (303)	0.170 (0.376)	740 (4594)	740 (880)	482 (756)
p3	123 (135)	167 (132)	224 (219)	0.173 (0.378)	563 (3235)	563 (537)	339 (470)
p4	120 (135)	165 (131)	242 (266)	0.145 (0.352)	568 (3172)	568 (601)	327 (487)
p5	123 (139)	167 (134)	236 (247)	0.178 (0.383)	729 (4685)	729 (775)	493 (689)

For a definition of the reading measures see Table 12. The Text column shows the unique text identifiers as introduced in Table 3

In some trials, the participant did not only read the text but also scanned the screen without reading. Such sequences of fixations can be easily distinguished from eye movements reflecting reading by visual inspection (automatized approaches exist, but are less accurate, see Biedert, Hees, Dengel, and Buscher 2012). Whenever such sequences of non-reading fixations occurred at the beginning or the end of a trial, they were deleted within the process of correcting the fixation-to-character mapping. In addition, very short fixations that looked like optical artifacts were deleted. All three stages of the data up to this point, the converted, human readable raw data, the originally computed uncorrected fixations and the manually corrected fixation data (corrected fixation location and deleted non-reading sequences) are made available in the data repository.

#### Computation of word-level reading measures

From the fixation data, various reading measures commonly used in reading research were computed for each text and reader. Each word (defined by the surrounding white spaces) was considered one area of interest for all measures except for *landing position* which was based on the characters within a word. Fixations on the punctuation marks were considered to belong to the preceding word by default and to the following word in case of opening parentheses or opening quotation marks. Definitions of the various measures are provided in Table 12 and an overview over the values of a few representative reading measures can be found in Tables 13 and 14. Note that several subsets of these measures are linearly dependent.

**Table 14:** Mean and standard deviation (in parentheses) of a selection of commonly used reading measures **for expert reading and non-expert reading** (see Fig. 1 for a definition of expert reading, and Table 12 for a definition of the reading measures)

Expert Reading	SFD	FFD	FPRT	FPReg	RPD_inc	TFT	RRT
non-expert reading	119 (136)	167 (133)	251 (313)	0.16 (0.37)	663 (4135)	663 (752)	412 (620)
expert	118 (136)	165 (133)	238 (265)	0.16 (0.37)	566 (3259)	566 (640)	328 (541)

### Summary of the available features

After all the data was annotated and pre-processed, PoTeC has different features available at different levels (word-, text-, reader-, fixation- and trial-level)^11^. *1) Word-level features*: All word-level features, which include manual and (semi)-automatically computed or estimated tags, are defined in Tables 4–8. *2) Text-level features*: Each text was given a unique identifier encoded as textual feature explicitly encoding the text domain. In addition to the text itself, the text’s domain, the comprehension questions, all the answer options, and the encoding of the correct answers to all questions are text-level features. *3) Reader-level features*: The data collected through the demographic questionnaire and a unique reader identifier are reader-level features as well as the level of studies and the reader discipline. The order of the stimuli texts and the order of the answer options for each of the questions are reader-level features and the average response accuracy over all questions for each question type. *4) Fixation-level features*: A chronological fixation index, fixation duration, previous saccade duration, next saccade duration, and the area of interest are available fixation-level features. In addition, whether or not the fixation has been manually corrected, the original chronological fixation index, the uncorrected fixation location and the uncorrected area of interest are added as features. *5) Trial-level features*: For each text read by a participant, the response accuracy for all questions and the mean response accuracy for all text and all background questions are provided. In addition, a feature was added to each trial that encodes whether this specific trial is *expert reading* or not (see Fig. 1). In addition, trial-level features include the word-level reading measures that have been computed for each text and reader as described in Section “Computation of word-level reading measures”.

The fixation data and reading measures are processed further and merged with different features of the corpus material on different levels to facilitate a simple use of the data. Note that these steps do not add any additional information but combine and merge the existing corpus data in such a way that common analyses of eye movement data for both psycholinguistic as well as NLP use cases are simplified and therefore encouraged.

As the fixation data is originally only associated with the index of the fixated character, it is further processed to explicitly include the fixated character and word. Also included are word-level features of the fixated word as well as most trial-, session-, reader-, and text-level features in addition to the fixation-level features. The resulting data representation is then referred to as the scanpath for this reader and text, which represents the chronological order of the eye movements on the text. The reading measures are merged with all the remaining reader-, text-, trial-, and session-level features that had not been added before, as well as the remaining word-level features (i.e., the various word-level tags).

Both the reading measures data and the scanpaths are made available in a format that includes only the most important features and in an extended format that includes all available features merged with the eye movement data (compare Table 11). Note that those two data representations comprise the same data in two very different formats with the scanpath data representing one fixation per line in chronological fixation order and the reading measures data representing the eye movement data at the word-level with one line representing one word in the order of the text.

## Accessing the data

PoTeC consists of various different types of data which include on the one hand many large data files and on the other hand different Python scripts. To account for the differences in these data formats, two channels have been chosen to share the data. The data files are stored in an Open Science Framework (OSF) repository: https://osf.io/dn5hp/. The code is stored in a GitHub repository: https://github.com/DiLi-Lab/PoTeC. The GitHub repository can be cloned locally and the data files can be automatically downloaded using a Python script.^12^ Alternatively, the data files can be manually downloaded from OSF.

The stimulus texts are protected by copyright and cannot be published without buying a respective license. As the reading data relies on having access to the full texts, we acquired licenses^13^ to be able to publish the texts in full length as they were used for the experiment. The texts can be downloaded from our website.^14^ The questions are created by ourselves and can be accessed via our GitHub repository.^15^

**Fig. 3:**
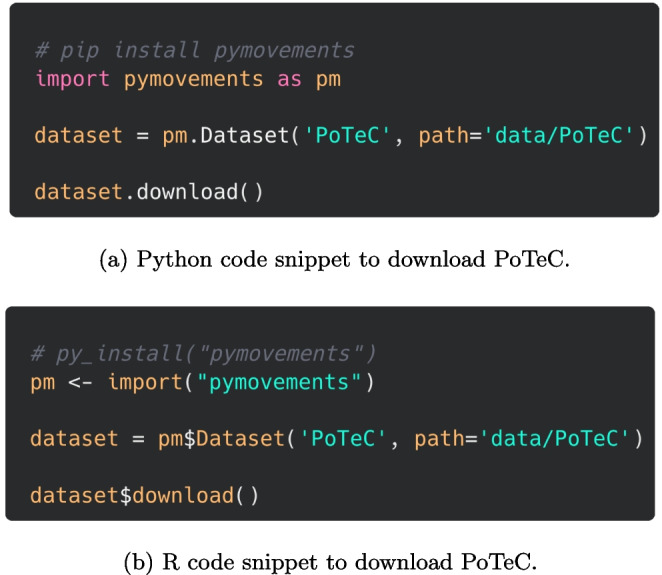
If pymovements has been installed locally, PoTeC can be easily downloaded to a custom location using the code snippet for Python or R

Additionally, for the users’ convenience, PoTeC has been integrated into the Python package pymovements^16^ (Krakowczyk et al., 2023). The package allows for downloading the data directly within a Python or an R script (see Fig. 3).^17^ Moreover, pymovements provides methods to further process the eye-tracking data. For example, besides many other functionalities, it provides various methods for gaze event detection including velocity-based (Engbert & Kliegl, 2003) as well as dispersion-based saccade or fixation detection algorithms (Salvucci & Goldberg, 2000), or a range of methods for visualizing eye-tracking data.

## Usage of the data

The data has already been used for different research purposes. For example, Krakowczyk et al. (2023) used PoTeC to develop explainable artificial intelligence (XAI) methods for analyzing deep neural networks processing eye-tracking data. Hollenstein, Pirovano, Zhang, Jäger, and Beinborn (2021) fine-tuned large language models on different eye-tracking corpora including PoTeC to better understand to what extent the representations learned by large language models are comparable to human reading behavior. Makowski, Jäger, Abdelwahab, Landwehr, and Scheffer (2019) used PoTeC for biometric identification, and the prediction of reading comprehension. Very recently, the corpus has been used to study expert and non-expert reading (Škrjanec, Broy, & Demberg, 2023).

## Analyses of the data

### Reading time analysis

We have already illustrated a few past use cases for PoTeC in Section “Usage of the data”. In the following, we present a series of analyses on a range of reading measures (first-pass reading time, total fixation time, re-reading time, first-pass regressions) to assess the effects of the word- and sentence-level features as well as the impact of expertise on eye movements. More specifically, we deployed hierarchical (generalized) linear-mixed models to:

1. explore expert and non-expert reading behavior (see Fig. 1 for a visual representation of what we refer to as *expert reading*),
2. assess the effect of different word- and sentence-level features such as word length, surprisal, and lexical frequency, as well as whether the word represents an expert term, whether it was read in the expert reading condition (see Table 15), and the reader discipline.

We modeled each word-level reading measure *y* using age (continuous), expert reading (binary; 1=stimulus text is read by a *graduate* student of the *discipline* that constitutes the *text domain*, 0=else; see Fig. 1), expert technical term^18^ (binary; 1=expert technical term that is not generally understandable, 0=else), reader discipline (binary; 1=physics, 0=biology; the reader’s discipline of studies), word length (in number of characters), log-lemma frequency^19^, lexicalized surprisal^20^, as well as the interaction of expert reading with reader discipline, word length, log-lemma frequency and surprisal as predictors, formalized as follows:

$$\begin{aligned} \hat{y}_{ij} =&g(\beta _0 + \beta _{0i} {+ \beta _{1}~\texttt {age}_i} + \beta _{2} ~ \texttt {expert reading}_{i}\\ &+ \beta _{3} ~ \texttt {reader discipline}_{i}+ \\ &\quad \beta _{4} ~ \texttt {reader discipline}_{i} * \texttt {expert reading}_{i}+ \\ &\quad \beta _{5} ~ \texttt {expert technical term}_{i}\\ &+ \beta _{6} ~\texttt {word length}_{j} + \\ &\quad \beta _{7} ~ \texttt {expert reading}_{i} * \texttt {word length}_{j}\\ &+ \beta _{8} ~\texttt {log-lemma frequency}_{j} + \\ &\quad \beta _{9} ~ \texttt {expert reading}_{i} * \texttt {log-lemma frequency}_{j}\\&+ \beta _{10} ~\texttt {surprisal}_{j} + \\ &\quad \beta _{11} ~ \texttt {expert reading}_{i} * \texttt {surprisal}_{j}) \end{aligned}$$

**Table 15:** Available features of PoTeC at different levels

Feature level	Features
Reader-level	Unique reader identifier, age, handedness, gender, how many hours of sleep the reader had the night before the experiment, grade of the German university entrance diploma, whether or not the reader wears glasses, whether or not the participant consumed alcohol within 24 h before the experiment start, **level of studies**, **reader discipline**, the order of texts in this session, and the order of answer options for all question.
Text level	Unique text identifier, text, questions, answer options, the correct answer for all questions, and **text domain**.
Trial-level	Response accuracy for all questions for the respective text, mean response accuracies for text and background questions for the text, and whether or not the trial is an example of **expert reading** (i.e., the text is read by a graduate whose discipline of studies equals the text domain) and the word level reading measures defined in Table 12.
Word level	All features defined in Tables 4–8.
Fixation level	Chronological fixation index, fixation duration, previous saccade duration, next saccade duration, whether or not the fixation has been manually corrected, the original chronological fixation index, fixated character and word, line index of the fixated character and the character index in the line.

Note: The features marked in bold are either the experimental factors or features based on the experimental factors

where $\hat{y}_{ij}$ refers to the eye-tracking reading measure of subject *i* for the *j*th word in the stimulus corpus across all texts. $\beta _0$ represents the global intercept, and $\beta _{0i}$ the random intercept for subject *i*. $g(\cdot )$ denotes the linking function: $g(z)=\ln \frac{z}{1-z}$ for the binary measure (first-pass regression) with $\hat{y}_{ij}$ following a Bernoulli distribution, which corresponds to logistic regression; and the identity function for the remaining continuous measures with $\hat{y}_{ij}$ following a log-normal distribution, which corresponds to linear regression on a log-transformed dependent variable. We ran each model for 6000 iterations using standard priors, provided in Appendix F. Note that all non-factorial predictors were standardized. We additionally re-ran the same analysis with down-sampled data where we removed the oldest and youngest participants from the data set (N_DS_=55) to obtain more homogeneous age groups. This is to assess whether age is confounded with the level of studies as graduate students are on average older than undergraduate students (see Table 9).

#### Results

**Fig. 4:**
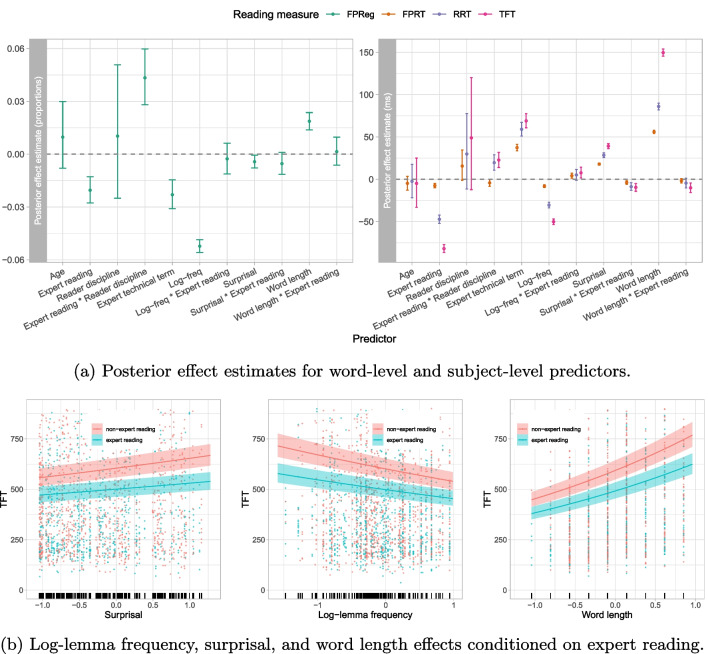
**a** Posterior effect estimates (mean and 95% credible interval) of word- and subject-level predictors on first-pass regressions (FPReg) in change in proportion, first-pass reading times (FPRT), re-reading times (RRT) and total fixation times (TFT) in milliseconds. Predictors include *age*, *expert reading*, *reader discipline*, the interaction of *expert reading* with *reader discipline*, *expert technical term* as well as *log-lemma frequency*, *surprisal* and *word length* and the interactions of *log-lemma frequency*, *surprisal*, and *word length* with *expert reading*. **b** Illustration of *z*-score normalized log-lemma frequency, surprisal, and word length conditioned on expert reading. The *line* represents mean predicted total fixation times for expert and non-expert trials, the *shaded area* represents a 95% credible interval

The results of the reading time analysis are presented in Fig. 4. With respect to the effects of word features on reading times, we found various expected effects. Higher lemma-frequency facilitates processing (shorter reading times, fewer regressions), while increased word length is associated with increased processing effort (longer reading times, more regressions). Similarly, high surprisal is associated with longer reading times. Moreover, participants show increased reading times on expert technical terms. We found that expert reading is characterized by shorter reading times, most strongly observed in total fixation times, and fewer regressions. Additionally, the positive interaction coefficient between expert reading and reader discipline suggests that physics graduates make more regressions and exhibit higher total-fixation and re-reading times during expert reading (i.e., when reading a text in the domain of their discipline of studies) than biology graduates. These effects, in particular for total fixation and re-reading times are very substantial, which is indicated by large posterior mean estimates and narrow credible intervals (the interaction coefficient $\hat{\beta }_4$ for total fixation times: $\hat{\beta }_4=22.76\text {ms}~[13.95\text {ms};31.70\text {ms}]$, and for re-reading times: $\hat{\beta }_4=19.65\text {ms}~[10.69\text {ms};28.89\text {ms}]$). As graduate students are on average older than undergraduate students (see Table 9), we analyzed to what extent the level of studies is confounded by age. If the level of studies was confounded by age, the effects we observe for expert reading could be caused by age, rather than the level of studies of the readers, as expert reading only includes graduate, that is, on average older students. Note that non-expert reading, on the other hand, is not generally characterized by lower age of individual readers, as non-expert reading not only includes undergraduate but also graduate students who are reading a text from a domain which is not their discipline of studies (see Fig. 1). As the upper panels in Fig. 4 show, we did not find an effect of age on any of the investigated reading measures. This additionally validates the observed effects of the expert reading predictor. The results of the down-sampling analysis are presented in Appendix H and confirm that the effects on reading times are caused by expert reading regardless of age.

### Analysis of the response accuracy for the text comprehension and background questions

PoTeC can be used to study the text comprehension and general background knowledge of the readers based on their discipline of studies and their level of studies. As specified in Section “Comprehension & background questions”, all questions have been created by experts in the respective domain of the text. The mean response accuracies of all groups of participants (according to the study design presented in Fig. 1) have been analyzed for each set of questions, that is, biology text and background questions, and physics text and background questions.

While the background questions are meant to test the domain knowledge of the participants in the area of the text’s topic (e.g., not biology in general but molecular biology), the text questions should allow for drawing conclusions about the readers’ text comprehension unrelated to their domain knowledge.

#### Results

The results of the response accuracies of the eye-tracking experiment are presented in Table 16. The mean response accuracies for expert reading trials for both text and background questions are significantly higher than for most non-expert trials for the same set of questions (e.g., physics graduates compared to biology undergraduates answering physics text questions). This indicates that expert readers are not only characterized by more background knowledge on the topic but that being an expert in a domain also facilitates understanding a text in the domain of expertise. In general, readers performed worse on background questions than on text questions, and worse on the physics questions than on the biology questions (except for physics graduates).

**Table 16:** Mean response accuracy (standard deviation and number of readers in parentheses) of the respective group of participants for each text domain for the text and background questions

	

Values marked in bold denote expert reading trials (see Fig. 1) and values in italics denote the highest mean response accuracy for that set of questions (i.e., biology or physics text or background questions). The asterisk and the brace denote that the mean response accuracy of the expert reading trials is significantly higher compared to the respective non-expert reading trials ($$***: p\le 0.001$$, $$**: p\le 0.01$$, $$*: p\le 0.05$$; unpaired one-tailed *t* test)

### Validation of the text comprehension questions

In order to validate whether the text questions could only be answered by reading the respective text and not by domain knowledge, or simply by guessing, we performed an additional online survey with readers who have not read the texts, but were asked to answer the text questions without the text. If the text questions cannot be answered without the text, any analyses investigating the responses to the text questions actually assess the participants’ text comprehension rather than their domain or even world knowledge.

We recruited graduate biology or physics students for the survey (i.e., they have already successfully received at least a bachelor’s degree in one of those fields). A third group of participants had no degree in either of the two subjects. Note that this allows for identifying graduates in both domains as for the eye-tracking experiment, but does not allow for identifying undergraduates in the same way which limits the comparison of non-expert reading. All participants were required to be German native speakers. A short participant questionnaire was presented before the questions to confirm these characteristics. The questions were presented in pseudo-randomized order. All questions for one text were presented in one block. The order of the questions within one block and the overall order of the blocks were randomized. All answer options were randomized as well for each participant. The participants received a compensation of 10 EUR upon completion of the survey.

To verify that participants were still paying attention to the questions, an attention check question was asked (refer to Appendix G to find all the questions) after six questions (i.e., after all text questions of two texts have been asked) that was supposed to be straightforward to answer given very basic world knowledge. All participants who did not answer all attention checks correctly were excluded from the analysis, as well as participants who hold a bachelor’s degree in both disciplines. In total, 40 participants took part in the survey of which five were excluded.

**Table 17:** Mean response accuracy (standard deviation and number of readers in parentheses) of the respective group of participants for answering the text questions without having read the text they belong to

Text	Level of	Reader	Mean response
domain	studies	discipline	accuracy on text questions
Biology	graduate	Biology	0.35 (*std*=0.07, *N*=9)*
		Physics	0.32 (*std*=0.10, *N*=13)*
	other^a^	other^a^	0.30 (*std*=0.10, *N*=$$13)*$$
Physics	graduate	Biology	0.21 (*std*=0.07, *N*=9)*
		Physics	0.19 (*std*=0.07, *N*=13)*
	other^a^	other^a^	0.21 (*std*=0.08, *N*=$$13)*$$

These participants were recruited online

Note: The asterisk denotes that this result is significantly less accurate ($p\le 0.001$; unpaired one-tailed *t*-test) than the respective group of participants that read the texts before answering the questions (see Table 16; e.g., the two groups of biology graduates answering biology text questions were compared; as the participant selection criteria were slightly different for the online survey, the “other” group was compared to all undergraduates answering the text questions from the respective domain)

^a^ The participants are neither physics nor biology graduates but otherwise it is unknown what degree and background they have

#### Results

An overview of the accuracy of the responses from the online survey is presented in Table 17. An accuracy of 0.25 is equivalent to random guessing as all questions have four answer options. The results show that 1) the response accuracies across all groups in the online survey are very similar for graduates as well as for participants with a different background, 2) they are close to random guessing and 3) for all groups, the response accuracies are significantly lower compared to the ones where participants were answering the questions after reading the texts (Table 16). Our comparison between the participant groups that took part in the eye-tracking experiment and the respective groups in the online survey supports our assumption that simply guessing the right answers without reading the texts is very difficult. Both expert and non-expert reading clearly benefits from reading the text before answering the text questions. This further means that the text questions are suitable for assessing the readers’ text comprehension.

## Summary & Conclusion

We presented PoTeC, a naturalistic German eye-tracking-while-reading corpus. PoTeC is the first eye-tracking dataset using a novel experiment design where the readers’ expertise has been manipulated within-subjects. That is, experts and non-experts from two different disciplines read texts from both disciplines. The stimulus corpus has been comprehensively linguistically annotated with a wide range of features on different linguistic levels obtained through manual annotation, extracted from databases, or computed by state-of-the-art computational models. The eye movement data is made available at *all* pre-processing stages with maximal flexibility for the user to choose which version of the data is useful for their use case (e.g., areas of interest at character- and word-level, uncorrected and corrected fixation data, scanpath data, raw data samples, etc.). The data is released with all the code that has been written for annotating or pre-processing the data which means that the entire pipeline can be fully reproduced and the code can be adapted to other use cases. Extensive demographic data has been collected for the participants and reading comprehension scores have been collected through comprehension questions and, in addition, the readers’ background knowledge has been assessed through background questions on the different topics of the stimulus texts. The data and code are made available in a way that creates maximal transparency, maximally increases the re-usability of the data and the code, and makes the data accessible in a user-friendly way using easy-to-understand data formats. In addition, the data has been integrated into an existing open-source software^21^ for the processing of eye movements that can be used in Python and R.

Given the different features described above, we envision different use cases of the data: 1) PoTeC can be used to study within-subject expert and non-expert reading patterns. As our exploratory analyses have shown, expert reading behavior can be characterized by shorter reading times when compared with non-expert reading. PoTeC is the first eye-tracking-while-reading dataset to allow such analyses. 2) All of these features can be used to train computational models to learn how to distinguish expert readers from non-expert readers. That is, models could be trained, for example, to infer the reader’s domain expertise when presented with a scanpath on a particular text. Such models could be used to conduct tests in digital learning scenarios to assess the learner’s progress. 3) The naturalistic reading data can be leveraged for NLP purposes without specifically studying expert reading. 4) The aim of the corpus is not only to make all the data available but also to publish any scripts and tools that were used to preprocess the data. This should increase transparency and foster high-quality data as it allows for analyzing not only the data but also the data collection and preprocessing pipeline which can help to continuously improve future data collections and algorithms to preprocess eye-tracking data. 5) Making the uncorrected and the manually corrected fixation data available allows for investigating how fixation data can be corrected automatically. PoTeC is the first corpus to make this kind of data available. In particular, the data can be used to train computational models to automatically correct fixation data as the manual correction is a *very* time-consuming endeavor but in practice often necessary and often part of preprocessing pipelines.

There exist many more use cases such as: creating models based on raw data, analyses of good and poor reading comprehension, inference of the reader’s reading comprehension, inference of whether a text is difficult for a reader, or whether it is a text from their domain of expertise. Our vision is that the corpus can be used for all of the above-mentioned use cases and many more that go beyond psycholinguistics, cognitive reading research or NLP. Finally, the work we described aims to encourage further eye-tracking data collections that can complement the existing data.

## Open Practices Statement

Materials and code are available in a GitHub repository at https://github.com/DiLi-Lab/PoTeC. The large data files are stored at https://osf.io/dn5hp/, but can be downloaded via the GitHub repository and pymovements: https://pymovements.readthedocs.io/en/v0.18.0/reference/pymovements.datasets.PoTeC.html. None of the reported studies were preregistered.

## Figures and Tables

**Fig. 5:**
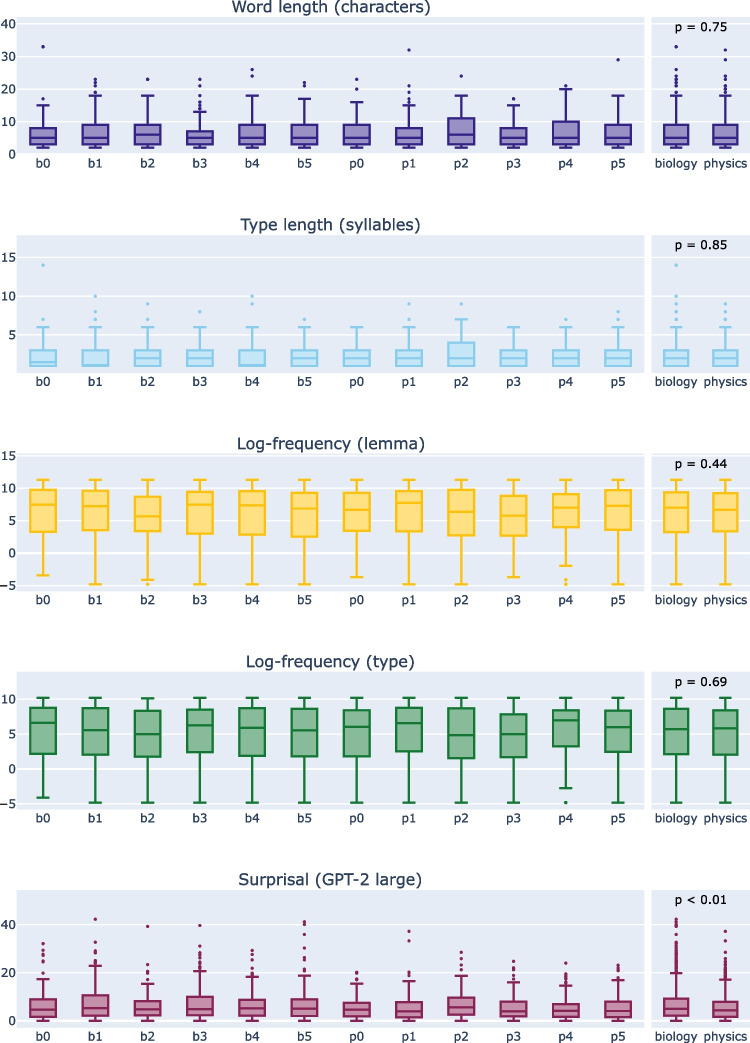
Domain- and text-specific summary of word length (character and syllable), log frequency (lemma and type) and surprisal (GPT-2 large estimated with left-hand sentence context) across texts. The upper/lower frame of the boxes represent the 25% and 75% quartiles of the data distribution, the line inside the box represents the median, and the whiskers mark the max/min point within all data points that have not been classified as outliers. The interquartile range (IQR; length of the box from the upper to the lower frame) is multiplied by 1.5. The box is extended by this length on both sides. All data points outside of this extended range are outliers (represented as dots). The distributions of each characteristic of the two domains have been compared using an unpaired *t*-test assuming non-equal variance

**Fig. 6:**
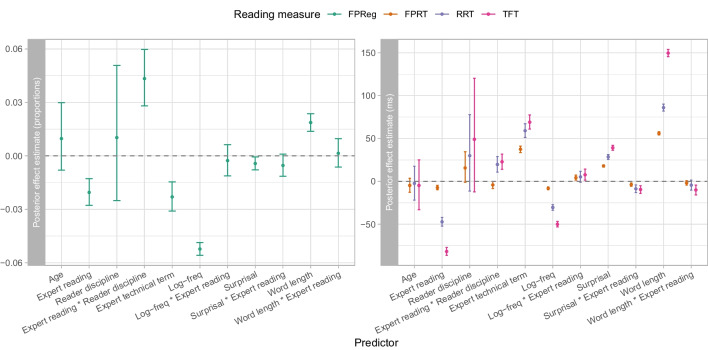
Posterior effect estimates in change in proportion (left) and milliseconds (right) (mean and 95% credible interval) for predictors *age*, *expert reading*, *reader discipline*, the interaction of *expert reading* with *reader discipline*, *expert technical term* as well as *log-lemma frequency*, *surprisal* and *word length* (number of characters) and the interactions of *log-lemma frequency*, *surprisal*, and *word length* with *expert reading*

## Data Availability

Data and materials are available in a GitHub repository at https://github.com/DiLi-Lab/PoTeC. The large data files are stored at https://osf.io/dn5hp/. The stimulus texts can be downloaded from our website https://www.cl.uzh.ch/en/research-groups/digital-linguistics/resources/potec.html.

## Appendix A Eye-tracking-while-reading datasets

The tables below present an extensive overview on existing eye-tracking-while-reading datasets. Note that all information in the tables is taken from the original papers and not all authors report the same information.

**Table 18:** Naturalistic eye-tracking-while-reading corpora for text passages in **English** (part I)

**Stimuli**	**Participants**	**Additional characteristics**
**SB-SAT** (Ahn et al., 2020)		
4 SAT passages taken from practice tests for reading comprehension	95 undergraduate students	Subjective difficulty ratings **Eye-tracker**: EyeLink 1000, **Sampl. Freq.**: 1000 Hz
**Passage reading** (Parker, Kirkby, & Slattery, 2017)		
40 text passages constructed around target words which will either appear in parafovea or not	48 native speakers ($\bar{a}$: 26.48 (14.83))	Constructed specifically to study parafoveal viewing. **Eye-tracker**: EyeLink 1000, **Sampl. Freq.**: 1000 Hz
**Provo Corpus** (Luke & Christianson, 2017)		
55 short texts from various different sources ($\# w$: 2750, $\overline{\# w}$: 50)	84 native speakers	Includes human predictability norms **Eye-tracker**: EyeLink 1000 Plus, **Sampl. Freq.**: 1000 Hz
**MQA-RC** (Sood et al., 2020)		
32 movie plots with comprehension questions including results from computational models answering the questions (200–250 words)	23 native speakers	The participants had to answer comprehension questions in different settings (e.g., with/without plot). **Eye-tracker**: Tobii, **Sampl. Freq.**: 600 Hz
**ASD Data** (Yaneva, 2016)		
27 texts from various sources ($\# w$: 4212, $\overline{\# w}$: 156 (49.94))	108 native speakers, 56 diagnosed with ASD and a control group ($\bar{a}$: 33.73 (8.36))	Groups of participants read disjoint subsets of the texts. Eye-tracker: Gazepoint GP3, **Sampl. Freq.**: 60 Hz
**GazeBase - Reading Task** (Griffith, Lohr, Abdulin, & Komogortsev, 2021)		
18 passages from the same poem with a maximum of 60 s to read two texts	322 participants, not all of them read all passages	Reading was spread over 3 years **Eye-tracker**: EyeLink 1000, **Sampl. Freq.**: 1000 Hz
**InteRead** (Zermiani et al., 2024)		
One excerpt from Arthur Conan Doyle’s “The Adventure of the Speckled Band”, 28 pages ($\overline{\# w}$: 154 (22.3) per page)	57 native adults ($\bar{a}$: 27.51 (5.55))	The reading process was interrupted at several previously chosen pages **Eye-tracker**: Tobii Pro Spectrum, **Sampl. Freq.**: 1200 Hz

All information in the table is taken from the original papers and not all authors report the same information

Abbr.: $\# w$ = total number of words; $\overline{\# w}$ = mean number of words per stimulus text with standard deviation in parentheses; $\bar{a}$ = mean age of participants with standard deviation in parentheses

**Table 19:** Naturalistic eye-tracking-while-reading corpora for text passages in **English** (part II)

**Stimuli**	**Participants**	**Additional characteristics**
**OneStopQA Eye-Tracking** (Malmaud, Levy, & Berzak, 2020)		
30 Guardian articles, each in an advanced difficulty version ($\# w$: 3858, $\overline{\# w}$: 128.6) and an elementary version ($\# w$: 3369, $\overline{\# w}$: 112.3)	269 participants, each participant read ten articles	One question per text was shown prior to reading or after reading the text **Eye-tracker**: EyeLink 1000 Plus, **Sampl. Freq.**: 1000 Hz
**CFILT - Coreference** (Cheri, Mishra, & Bhattacharyya, 2016)		
22 texts (less than ten sentences each text)	14 non-native participants: 2 are expert linguists (age: 47–50), 12 (post-)graduates (age: 20–30)	Participants annotated coreferences in the text. **Eye-tracker**: EyeLink 1000 Plus, **Sampl. Freq.**: 500 Hz
**CFILT - Scanpath** (Mishra, Kanojia, Nagar, Dey, & Bhattacharyya, 2017b)		
32 paragraphs from (simple) Wikipedia (50–200 words each)	16 non-native participants: 3 are expert linguists (age: 47–50), 13 (post-)graduates (age: 20–30)	Participants annotated texts for the effort to read them. **Eye-tracker**: EyeLink 1000 Plus, **Sampl. Freq.**: –
**CFILT - Essay Grading** (Mathias, Murthy, Kanojia, Mishra, & Bhattacharyya, 2020)		
48 essays (max. words per essay: 250)	8 fluent English speakers ($\bar{a}$: 25)	Participants graded the essays after reading. **Eye-tracker**: EyeLink 1000, **Sampl. Freq.**: 500 Hz
**CFILT - Text quality** (Mathias et al., 2018)		
30 texts from different sources (ca. 200 words in each text)	20 fluent English speakers (age: 20-25)	Participants annotated the text quality based on three given properties (organization, coherence and cohesion). **Eye-tracker**: EyeLink 1000, **Sampl. Freq.**: 500 Hz
**MECO-L2** (Kuperman et al., 2023)		
12 encyclopedic texts originally designed for English language testing ($\# w$: 1653, $\overline{\# w}$: 137.8 (25.3))	543 non-native participants ($\bar{a}$: 23.4), L1^a^: nl, en, el, de, he, it, ru, es, tr, no, et, fi	**Eye-tracker**: EyeLink Portable Duo, 1000 or 1000 Plus, **Sampl. Freq.**: 1000 Hz
**MECO-L2 – Wave 2** (Kuperman et al., 2024)		
12 encyclopedic texts originally designed for English language testing ($\# w$: 1653, $\overline{\# w}$: 137.8 (25.3))	661 non-native participants ($\bar{a}$: 22.3), L1^a^: en, de, es, tr, no, hi, da, ru, sr, eu, is, zh, pt	**Eye-tracker**: EyeLink Portable Duo, II, 1000 or 1000 Plus, **Sampl. Freq.**: 1000 Hz

All information in the table is taken from the original papers and not all authors report the same information

Abbr.: $\# w$ = total number of words; $\overline{\# w}$ = mean number of words per stimulus text with standard deviation in parentheses; $\bar{a}$ = mean age of participants with standard deviation in parentheses

^a^ Language abbr. (ISO-639-1 Code): nl: Dutch, en: English, el: Greek, de: German, he: Hebrew, it: Italian, ru: Russian, es: Spanish, tr: Turkish, no: Norwegian, et: Estonian, fi: Finnish, da: Danish, sr: Serbian, is: Icelandic, zh: Chinese, eu: Basque, pt: Portuguese, hi: Hindi

**Table 20:** Naturalistic eye-tracking-while-reading corpora for single sentences in **English**

**Stimuli**	**Participants**	**Additional characteristics**
**CELER** (Berzak et al., 2022)		
156 sentences from the Wall Street Journal ($\# w$: 900, $\overline{\# w}$: 11.3)	365 participants: 69 English L1 and 296 English L2 ($\bar{a}$: 27.3 (6.8))	The L2 readers have five different L2 backgrounds and half of the stimuli are uniquely read by one participant. **Eye-tracker**: EyeLink 1000 Plus, **Sampl. Freq.**: 1000 Hz
**CFILT Sarcasm** (Mishra, Kanojia, & Bhattacharyya, 2016)		
1000 sentences, 350 are labeled as sarcastic and 650 as non-sarcastic	7 non-native English speakers	Participants were asked to label the sentences as either positive or negative. **Eye-tracker**: EyeLink 1000, **Sampl. Freq.**: 500 Hz
**CFILT Sentiment** (Joshi, Mishra, Senthamilselvan, & Bhattacharyya, 2014)		
1059 movie reviews or twitter posts	5 participants	Participants were asked to label the sentiment of the sentences as either positive, negative or objective. **Eye-tracker**: Tobii TX 300, **Sampl. Freq.**: 300 Hz
**UCL Corpus** (Frank, Fernandez Monsalve, Thompson, & Vigliocco, 2013)		
205 sentences selected from free online novels ($\# w$: 2399, $\overline{\# w}$: 13.7 (6.36))	43 native speakers ($\bar{a}$: 25.8)	The dataset contains self-paced-reading data as well. **Eye-tracker**: EyeLink II, **Sampl. Freq.**: 500 Hz
**ZuCo 1** (Hollenstein et al., 2018)		
1107 Wikipedia sentences and movie reviews ($\overline{\# w}$: 19.54 (9.72))	12 native speakers ($\bar{a}$: 38 (9.8))	Co-registration of EEG data and each sentence was part of a block with a specific task. **Eye-tracker**: EyeLink 1000 Plus, **Sampl. Freq.**: 500 Hz
**ZuCo 2** (Hollenstein, Troendle, Zhang, & Langer, 2020)		
739 Wikipedia sentences ($\# w$: 15,138, $\overline{\# w}$: 20.5 (9.2))	18 native speakers ($\bar{a}$: 34 (8.3))	Co-registration of EEG data and each sentence was part of a block with a specific task. **Eye-tracker**: EyeLink 1000 Plus, **Sampl. Freq.**: 500 Hz

All information in the table is taken from the original papers and not all authors report the same information

Abbr.: $\# w$ = total number of words; $\overline{\# w}$ = mean number of words per stimulus text with standard deviation in parentheses; $\bar{a}$ = mean age of participants with standard deviation in parentheses

**Table 21:** Naturalistic eye-tracking-while-reading corpora for single sentences in different languages other than English

**L**	**Stimuli**	**Participants**	**Additional characteristics**
**Beijing Sentence Corpus** (Pan, Yan, Richter, Shu, & Kliegl, 2021)			
zh	150 from the People’s Daily newspaper ($\overline{\# w}$: 11.2 (1.6))	60 native speakers ($\bar{a}$: 22.0 (2.6))	The dataset includes human predictability norms. **Eye-tracker**: EyeLink II, **Sampl. Freq.**: 500 Hz
**Potsdam Sentence Corpus** (Kliegl et al., 2004, 2006)			
de	144 sentences constructed around target words ($\overline{\# w}$: 7.9)	222 native speakers (age: 16-84)	The dataset includes human predictability norms. **Eye-tracker**: EyeLink I / EyeLink II, **Sampl. Freq.**: 250 / 500 Hz
**Potsdam-Allahabad Hindi Eyetracking Corpus** (Husain, Vasishth, & Srinivasan, 2014)			
hi, ur	153 sentences from the Hindi-Urdu treebank ($\# w$: 2610, $\overline{\# w}$: 17.0)	30 participants	The same sentences were read in Hindi and Urdu script in two separate sessions. **Eye-tracker**: SMI iView X HED, **Sampl. Freq.**: 500 Hz
**Chinese Word Length Effect** (Zang, Fu, Bai, Yan, & Liversedge, 2018)			
zh	90 constructed sentences ($\overline{\# c}$: 19.0 (2.0))	30 native speakers ($\bar{a}$: 24.0 (2.0))	Sentences are rated for their naturalness; includes human predictability norms **Eye-tracker**: EyeLink 1000, **Sampl. Freq.**: – Hz
**Russian Sentence Corpus** (Laurinavichyute, Sekerina, Alexeeva, Bagdasaryan, & Kliegl, 2018)			
ru	144 selected sentences based on target words from the Russian National Corpus ($\# w$: 1362)	96 native speakers ($\bar{a}$: 24.0)	The dataset includes human predictability norms. **Eye-tracker**: EyeLink 1000 Plus, **Sampl. Freq.**: 1000 Hz
**RaCCooNS** (Frank & Aumeistere, 2023)			
nl	200 narrative sentences ($\# w$: 2783)	37 native speakers ($\bar{a}$: 26.2)	Co-registration of EEG data. **Eye-tracker**: EyeLink 1000 Plus, **Sampl. Freq.**: 1000 Hz
**Hong Kong Corpus of Chinese Sentence and Passage Reading** (Wu & Kit, 2023)			
zh	300 single-line sentences ($\# w$: 5250) and 7 multi-line passages ($\# w$: 4967) from newspaper articles	96 native speakers ($\bar{a}$: 26 (3.64))	**Eye-tracker**: EyeLink 1000, **Sampl. Freq.**: 1000 Hz
**Eye-movement Measures on Words in Chinese Reading** (Zhang et al., 2022)			
zh	7577 sentences ($\# w$: 8551, $\overline{\# c}$: 22.48)	1718 native speakers	**Eye-tracker**: EyeLink 1000, **Sampl. Freq.**: 1000 Hz
**TURead** (Acartürk et al., 2023)			
tr	192 sentences selected from existing Turkish corpora based on target words ($\overline{\# w}$: 15.3 (2.9)); 37 stimuli consist of 2 or 3 sentences	196 native speakers ($\bar{a}$: 22.7 (2.6))	Includes human predictability norms; both silent and aloud reading. **Eye-tracker**: EyeLink 1000, **Sampl. Freq.**: 1000 Hz

All information in the table is taken from the original papers and not all authors report the same information

Abbr.: L = language of stimuli (ISO-639-1): zh: Chinese, de: German, hi: Hindi, ur: Urdu, ru: Russian, nl: Dutch, tr: Turkish; $\# w$ = total number of words; $\overline{\# w}$ = mean number of words per stimulus text with standard deviation in parentheses; $\overline{\# c}$ = mean number of characters per stimulus text; $\bar{a}$ = mean age of participants with standard deviation in parentheses

**Table 22:** Naturalistic eye-tracking-while-reading corpora on text passages in different languages (except German and English), or on multilingual text passage corpora

**L**	**Stimuli**	**Participants**	**Additional characteristics**
**GECO** (Cop, Dirix, Drieghe, & Duyck, 2016)			
en, nl	Complete novel which is easy to read in English and Dutch ($\# w$ en: 54,364, $\# w$ nl: 59,716)	33 participants: 19 bilinguals (L1: nl, L2: en) ($\bar{a}$: 21.2 (2.2)) and 14 monolinguals (en) ($\bar{a}$: 21.8 (5.6))	Bilinguals read one half in Dutch, the other in English. **Eye-tracker**: EyeLink 1000, **Sampl. Freq.**: 1000 Hz
**GECO-CN** (Sui, Dirix, Woumans, & Duyck, 2022)			
zh, en	Complete novel in Chinese and English ($\# w$ en: 54,364, $\# w$ zh: 59,403)	30 bilinguals (L1: zh, L2: en) ($\bar{a}$: 25.3 (2.6))	Participants read one half in Chinese, the other in English. **Eye-tracker**: EyeLink 1000 Plus, **Sampl. Freq.**: 1000 Hz
**Gaze Behavior in Text Summarization** (Yi, Guo, Jiang, Wang, & Sun, 2020)			
zh	100 articles from public news websites each belonging to one out of ten categories ($\overline{\# c}$: 502)	50 participants ($\bar{a}$: 23.1 (1.1))	Participants were asked to summarize each text after reading. **Eye-tracker**: Tobii EyeTracking 4C, **Sampl. Freq.**: 100 Hz
**CopCo** (Hollenstein, Barrett, & Björnsdóttir, 2022; Reich, Deng, Björnsdóttir, Jäger, & Hollenstein, 2024)			
da	20 speech manuscripts ($\# w$: 34,897, $\overline{\# w}$: 1745)	57 participants: 25 native speakers, 19 dislexic native speakers and 13 L2 speakers (age: 21-62)	**Eye-tracker**: EyeLink 1000 Plus, **Sampl. Freq.**: 1000 Hz
**Reading Attention** (Li et al., 2018)			
zh	60 documents, each belongs to one out of 15 search queries	29 native speakers (age: 17–28) each participant read 15 documents (one for each query)	The participants were asked to judge the relevance of the document for the query. **Eye-tracker**: Tobii X2-30, **Sampl. Freq.**: – Hz
**Mental Simulation Corpus** (Mak & Willems, 2019)			
nl	3 literary short stories ($\# w$: 10,800, $\overline{\# w}$: 2600)	102 participants ($\bar{a}$: 23)	Participants answered questions that allow for studying their mental simulation during reading. **Eye-tracker**: EyeLink 1000 Plus, **Sampl. Freq.**: 500 Hz
**Dundee** (Kennedy et al., 2003, 2013)			
en, fr	Extracts of newspaper articles in French and English	20 participants, ten each language	Participants read the texts written in their native language. **Eye-tracker**: Dr Bouis Oculometer Eyetracker, **Sampl. Freq.**: 1000 Hz
**RastrOS** (Leal et al., 2022; Vieira, 2020)			
pt	50 paragraphs from journalistic, literary and popular science texts ($\overline{\# w}$: 2494, $\overline{\# w}$: 49 (7.47))	37 native speakers ($\bar{a}$: 22.2 (4.7))	Includes human predictability norms. **Eye-tracker**: EyeLink 1000, **Sampl. Freq.**: 1000 Hz

All information in the table is taken from the original papers and not all authors report the same information

Abbr.: L = language of stimuli (ISO-639-1): pt: Portugese, en: English, fr: French, nl: Dutch, zh: Chinese, da: Danish; $\# w$ = total number of words; $\overline{\# w}$ = mean number of words per stimulus text with standard deviation in parentheses; $\overline{\# c}$ = mean number of characters per stimulus text; $\bar{a}$ = mean age of participants with standard deviation in parentheses

**Table 23:** Self-paced naturalistic reading corpora for different languages for both single sentences and text passages

**L**	**Stimuli**	**Participants**	**Additional characteristics**
**UCL** (Frank et al., 2013)			
en	361 sentences selected from free online novels ($\# w$: 4946, $\overline{\# w}$: 13.7 (6.36))	117 psychology students ($\bar{a}$: 18.9)	The dataset contains eye-tracking data as well.
**Natural Stories Corpus** (Futrell et al., 2017)			
en	ten stories with a total of 485 sentences ($\overline{\# w}$ per sentence: 22.38), the texts have been edited to contain many hard-to-process constructions	181 participants, not all of them read all ten stories	

All information in the table is taken from the original papers and not all authors report the same information

Abbr.: L = language of stimuli (ISO-639-1): en: English; $\# w$ = total number of words; $\overline{\# w}$ = mean number of words per stimulus text with standard deviation in parentheses; $\bar{a}$ = mean age of participants with standard deviation in parentheses

## Appendix B Stimulus text examples

The following text^22^ (text id “p0”) is one of the physics texts as it was presented to the participants (Demtröder, 2014b) followed by the respective comprehension questions. Ein Zyklotron besteht aus einer flachen, zylindrischen Vakuumkammer zwischen den Polen eines Elektromagneten, der ein Feld in z-Richtung erzeugt. Die Kammer ist in zwei D-förmige Hälften aufgeteilt, zwischen denen eine Hochfrequenzspannung anliegt. Die von der Ionenquelle im Spalt zwischen den Kammern im Zentrum der Anordnung emittierten positiven Ionen werden auf die negative Kammerhälfte zu beschleunigt. Da im Inneren der Kammerhälften mit metallischen Wänden kein elektrisches Feld existiert, beschreiben die Ionen hier im Magnetfeld einen Halbkreis in der x-y-Ebene, dessen Radius durch die als Zentripetalkraft wirkende Lorentzkraft festgelegt und dessen Umlaufszeit unabhängig vom Radius ist. Wird die Hochfrequenz nun genau so gewählt, dass die Ionen nach Durchlaufen des Halbkreises immer zu einem Zeitpunkt wieder am Spalt ankommen, bei dem die richtige Polarität der Beschleunigungsspannung anliegt, nimmt ihre kinetische Energie bei Durchlaufen des Spaltes zu, ihre Geschwindigkeit wächst und daher auch der Radius des nächsten Halbkreises. Die Ionen durchlaufen deshalb eine spiralartige Bahn, die aus lauter Halbkreisen mit wachsenden Radien besteht, bis sie den Rand des Magnetfeldes erreicht haben und dort durch ein elektrisches Ablenkfeld extrahiert werden können. Text question 1: Warum muss die Umlaufszeit für einen Halbkreis unabhängig vom Radius sein? Damit die Ionen immer genau zum Feldwechsel am Spalt ankommen. Damit die Ionen sich nicht gegenseitig einholen. Damit die Ionen das Zyklotron nicht in z-Richtung verlassen. Damit sich der Radius der Ionenbahn erhöht. Text question 2: Wozu ist das elektrische Feld zwischen den Kammerhälften nötig? Um den Ionen kinetische Energie zu geben. Um die Ionen auf der Kreisbahn zu halten. Um die Ionen in der x-y-Ebene zu halten. Um die Ionen in z-Richtung zu beschleunigen. Text question 3: Wozu ist das magnetische Feld in den Kammerhälften nötig? Um die Ionen auf der Kreisbahn zu halten. Um den Ionen kinetische Energie zu geben. Um die Ionen in der x-y-Ebene zu halten. Um die Ionen in z-Richtung zu beschleunigen. Background question 1: Warum ist die Umlaufszeit für einen Halbkreis unabhängig vom Radius? Weil die Ablenkung im Magnetfeld nur von der Geschwindigkeit der Ionen abhängt. Weil die Ablenkung im Magnetfeld nicht von der Geschwindigkeit der Ionen abhängt. Weil der Radius sich quadratisch mit der Geschwindigkeit ändert. Weil der Radius nicht von der Geschwindigkeit abhängt. Background question 2: Was gilt für die kinetische Energie der Ionen nach Verlassen des Zyklotrons? Sie ist proportional zum Quadrat des Betrages des Magnetfeldes. Sie ist proportional zum Betrag des Magnetfeldes. Sie ist proportional zum Radius der Kammerhälften. Sie ist proportional zur Ladung des Teilchens. Background question 3: Was gilt für den Radius der Halbkreisbahnen? (Masse pro Ladung) mal (Geschwindigkeit pro Magnetfeldstärke). (Masse mal Ladung) durch (Geschwindigkeit mal Magnetfeldstärke). (Magnetfeldstärke pro Ladung) mal (Geschwindigkeit pro Masse). (Magnetfeldstärke mal Ladung) pro (Geschwindigkeit mal Masse). The following text^23^ (text id “b0”) is an example of one of the biology texts that was read by the participants (Ableitner, 2014). The text is followed by the respective text and background questions: Um das Vorhandensein der Polymerasekettenreaktion-Produkte feststellen zu können, verwendet man die Gelelektrophorese mit einer anschließenden Behandlung des Gels durch Ethidiumbromid. Bei dieser Methode wird die negative Ladung der DNA-Fragmente genutzt. Die DNA-Fragmente sind auf Grund der darin enthaltenen Phosphatreste negativ geladen. Anlegen eines elektrischen Feldes lässt die Polymerasekettenre-aktion-Produkte durch ein gelartiges Medium, zumeist Agarose, wandern. Die DNA-Fragmente wandern durch das “Agarose-Sieb” vom Minuspol zum Pluspol. Kleinere Fragmente wandern schneller durch das “Agarose-Sieb”, daher befinden sie sich in der Nähe zum Pluspol des Gels. Die größeren Fragmente wandern langsamer und bleiben in der Nähe zum Minuspol. Um DNA-Fragmente sichtbar zu machen, behandelt man das Gel mit einer wässrigen Lösung von Ethidiumbromid. Das Molekül des Ethidiumbromids ist planar und kann sich zwischen den Basen der DNA-Fragmente einlagern. Nur nach dem Entstehen eines solchen Komplexes und durch das Beleuchten mit UV-Licht kann man die Polymerasekettenreaktion-Produkte in Form von Banden sehen und fotografieren. Text question 1: Nach welchem Kriterium werden die DNA-Fragmente getrennt? Nach Länge. Nach Ladung. Nach Basensequenz. Nach Ethidiumbromideinlagerung. Text question 2: Wodurch sind die DNA-Fragmente negativ geladen? Durch die Phosphatreste im Rückgrat. Durch die negative Ladung der Base Adenin. Durch das Methylierungsmuster der DNA. Durch die Phosphoratome. Text question 3: Welche Eigenschaft der Agarose sorgt für die Trennung der DNA-Fragmente? Ihre dreidimensionale Netzstruktur. Ihre Zähflüssigkeit. Ihre negative Ladung. Ihre positive Ladung. Background question 1: Wie wird die absolute Länge der DNA-Fragmente bestimmt? Durch Positionsvergleich mit parallel mitlaufendem Marker. Durch die Dauer der Gelelektrophorese. Durch die absolute Positionen der Banden auf dem Gel. Durch die Intensität der Banden nach Anregung mit UV-Licht. Background question 2: Wie stellt man das Auflösungsvermögen des Gels ein? Durch die Agarosekonzentration. Durch die Stärke des elektrischen Feldes. Durch die Ethidiumbromidkonzentration. Durch die Wellenlänge des UV-Lichtes. Background question 3: Warum eignet sich Ethidiumbromid, um die Banden sichtbar zu machen? Es ist interkalierend. Es ist thermostabil. Es ist rot. Es ist chiral.

## Appendix C Text characteristics

Figure 5 presents an extensive overview over different text characteristics.

## Appendix D Calibration and validation overview

Table 24 presents an overview of the different validations and calibrations that were performed in each session including the eye-tracked, the average validation scores and the total number of calibrations/validations per session.

## Appendix E Participant instructions

This section contains 1) the instructions that were given to the participants before starting the eye-tracking-while-reading experiment and 2) the instructions that were presented to the participants of the online survey to test the validity of the text comprehension questions as outlined in Section “Validation of the text comprehension questions”. These are the original instructions in German.

### E.1 Instructions for eye-tracking-while-reading-experiment

Herzlich Willkommen zu unserer Eyetracking-Lesestudie! Wir danken dir ganz herzlich für deine Teilnahme!

In diesem Experiment wirst du insgesamt 12 kurze Texte zu physikalischen oder biologischen Themen lesen, die maximal eine Bildschirmlänge lang sind. Nach jedem Text beantwortest du inhaltliche Multiple-Choice-Fragen per Tastendruck. Während du liest, nimmt der Eyetracker die Bewegungen deiner Augen auf. Du bist berechtigt, das Experiment jederzeit ohne Angabe von Gründen abzubrechen. Bitte lies zunächst aufmerksam die folgenden Erläuterungen durch.

Der Ablauf für jeden Text ist wie folgt: An der Stelle, wo das erste Wort des Textes erscheinen wird, wird zunächst ein grauer Kalibrierungspunkt mit einem kleinen schwarzen Punkt in der Mitte erscheinen. Sobald du auf diesen winzigen schwarzen Mittelpunkt schaust, wird automatisch der Text angezeigt. Lies den Text ganz natürlich (ohne Stimme). Wichtig ist, dass du versuchst, den Text inhaltlich zu verstehen. Sobald du den Text fertiggelesen hast, schaue bitte auf den runden grünen Aufkleber in der unteren rechten Ecke des Bildschirms und drücke eine der beiden “weiter”-Tasten. Nun erscheinen nacheinander sechs Fragen zu dem Text. Alle Fragen sind Multiple-Choice-Fragen mit jeweils vier Antwortmöglichkeiten. Es ist immer nur eine Antwort richtig. Zum Auswählen deiner Antwort drückst du bitte die Taste mit der entsprechenden Nummer auf der Button-Box. Die Fragen sind teilweise recht schwierig und manche der Fragen prüfen auch Fachwissen, das über den eigentlichen Inhalt des Textes hinausgeht. Es ist sehr wichtig, dass du versuchst, alle Fragen trotzdem so gut wie möglich zu beantworten. Wenn du dir nicht sicher bist, wähle die Antwort, die du für am plausibelsten hälst.

Bevor das eigentliche Experiment beginnt, wirst du einen Übungstext mit Fragen sehen, damit du dich an den Ablauf gewöhnen kannst. Thema des übungstexts ist der Experimentablauf. Sollte noch etwas unklar sein, kannst du nach dem Übungstext die Versuchsleiterin fragen. Während des eigentlichen Experiments sollten nach Möglichkeit keine Fragen mehr gestellt werden. Bitte rücke den Stuhl nah an den Tisch heran und nimm eine aufrechte Haltung ein. Lege dein Kinn auf die dafür vorgesehene Halterung und lehne die Stirn an den Rahmen. Die Tischhöhe kann an deine Größe angepasst werden. Die Versuchsleiterin wird dir helfen, Tischhöhe und Kinnstütze anzupassen. Bitte lege deine Finger auf die Tasten der Button-Box, so dass die beiden Daumen auf den “weiter-Tasten” liegen und der linke Mittelfinger auf der 1, der linke Zeigefinger auf der 2, der rechte Zeigefinger auf der 3 und der rechte Mittelfinger auf der 4 liegt.

Wenn du deine Sitzposition gefunden hast, wird der Eyetracker auf deine Augen kalibriert. Auf dem Bildschirm werden graue Punkte erscheinen, in deren Mitte ein winziger schwarzer Punkt ist. Folge mit deinem Blick diesen winzigen schwarzen Punkten. Bitte fixiere jeden Punkt solange, bis er verschwindet. Bitte vermeide Kopfbewegungen während und nach der Kalibrierung. Solltest du den Kopf bewegen, muss die Kalibrierung leider wiederholt werden.

Du kannst immer dann eine Pause machen, nachdem du einen Text fertig gelesen und die Fragen dazu beantwortet hast. Sag einfach der Versuchsleiterin Bescheid! Falls du dich aus irgendeinem Grund schlecht fühlen solltest, kannst du natürlich jederzeit unterbrechen bzw. abbrechen. Vergütung: Du bekommst nach Beenden des Experiments mindestens 10EUR ausbezahlt. Darüber hinaus kannst du dir einen Bonus von bis zu 10EUR zusätzlich verdienen: Je mehr Fragen du richtig beantwortest, desto höher ist dein Bonus. Liegt deine Antwortgenauigkeit auf dem Zufallsniveau, bekommst du keinen Bonus. Den vollen Bonus bekommen Studienanfänger, wenn sie mindestens 43% aller Fragen richtig beantworten. Fortgeschrittene bekommen den vollen Bonus, wenn sie mindestens 63% aller Fragen richtig beantworten. Die Bezahlung erfolgt direkt nach dem Experiment in bar.

Viel Spaß!

**Table 24:** Number of nine-point calibrations and validations per session and the averages of the average and the maximal validation score for each validation in each session

ID	Type	Eye	$\overline{avg}$	$\overline{max}$	#	ID	Type	Eye	$\overline{avg}$	$\overline{max}$	#	ID	Type	Eye	$\overline{avg}$	$\overline{max}$	#
0	cal	R			16	34	cal	R			9	78	cal	R			14
	val	R	0.364	0.737	9		val	R	0.422	0.679	8		val	R	0.307	0.554	11
1	cal	R			20	35	cal	R			12	79	cal	R			14
	val	R	0.52	1.536	7		val	R	0.312	0.538	8		val	R	0.268	0.601	8
2	cal	R			25	36	cal	R			15	80	cal	R			12
	val	R	0.597	1.068	6		val	R	0.283	0.431	15		val	R	0.341	0.655	11
3	cal	R			13	37	cal	R			15	81	cal	R			13
	val	R	0.306	0.65	7		val	R	0.445	0.828	10		val	R	0.437	0.78	13
4	cal	R			6	38	cal	R			15	82	cal	R			14
	val	R	0.228	0.45	5		val	R	0.347	0.611	7		val	R	0.569	0.962	9
5	cal	R			10	39	cal	R			15	83	cal	L			19
	val	R	0.293	0.677	7		val	R	0.355	0.681	11			R			3
6	cal	R			12	40	cal	R			16		val	L	0.882	1.773	9
	val	R	0.289	0.489	9		val	R	0.463	0.88	10			R	2.37	5.045	2
7	cal	R			10	41	cal	R			14	84	cal	R			12
	val	R	0.251	0.566	8		val	R	0.343	0.744	7		val	R	0.334	0.876	7
8	cal	R			7	60	cal	R			9	85	cal	R			15
	val	R	0.287	0.528	6		val	R	0.475	1.135	4		val	R	0.364	0.701	14
9	cal	R			17	61	cal	R			27	87	cal	R			11
	val	R	0.579	1.739	7		val	R	0.518	0.922	8		val	R	0.274	0.572	8
10	cal	R			9	62	cal	R			22	90	cal	R			19
	val	R	0.294	0.508	5		val	R	0.34	0.58	13		val	R	0.688	1.693	6
12	cal	R			12	63	cal	R			17	91	cal	R			18
	val	R	0.384	0.754	5		val	R	0.5	0.817	9		val	R	0.378	0.637	6
13	cal	R			21	64	cal	R			21	92	cal	R			14
	val	R	0.375	0.664	8		val	R	0.372	0.6	14		val	R	0.261	0.486	12
14	cal	R			16	65	cal	R			16	93	cal	R			14
	val	R	0.416	0.789	10		val	R	0.499	0.967	14		val	R	0.304	0.549	8
15	cal	R			17	66	cal	R			21	94	cal	R			10
	val	R	0.407	0.981	14		val	R	0.266	0.566	13		val	R	0.443	0.813	7
16	cal	L			2	67	cal	R			22	95	cal	R			13
		R			20		val	R	0.441	1.301	9		val	R	0.188	0.422	5
	val	L	0.515	0.92	2	68	cal	R			11	96	cal	R			21
		R	0.743	1.425	13		val	R	0.285	0.493	6		val	R	0.278	0.482	14
17	cal	R			14	69	cal	R			17	97	cal	R			16
	val	R	0.315	0.61	13		val	R	0.508	1.021	9		val	R	0.282	0.515	12
18	cal	R			13	70	cal	R			15	98	cal	R			16
	val	R	0.409	0.774	10		val	R	0.305	0.528	12		val	R	0.454	0.906	7
19	cal	R			14	71	cal	R			16	99	cal	R			15
	val	R	0.409	0.756	10		val	R	0.291	0.52	12		val	R	0.355	0.725	6
20	cal	R			14	72	cal	R			8	100	cal	R			9
	val	R	0.369	0.67	10		val	R	0.267	0.532	6		val	R	0.412	0.799	9
22	cal	R			16	73	cal	R			8	101	cal	R			17
	val	R	0.574	1.351	9		val	R	0.316	0.616	7		val	R	0.53	1.069	14
23	cal	R			15	74	cal	R			14	102	cal	R			18
	val	R	0.426	0.784	8		val	R	0.353	0.581	7		val	R	0.522	1.163	15
30	cal	R			7	75	cal	R			10	103	cal	R			15
	val	R	0.34	0.613	3		val	R	0.318	0.48	6		val	R	0.5	0.993	10
31	cal	R			21	76	cal	R			19	104	cal	R			13
	val	R	0.532	1.002	9		val	R	0.342	0.793	10		val	R	0.395	0.982	10
32	cal	R			9	77	cal	R			8	105	cal	R			14
	val	R	0.267	0.503	6		val	R	0.453	0.82	7		val	R	0.438	0.818	9

ID denotes the unique reader ID

### E.2 Instructions for online survey to validate text comprehension questions

In dieser Studie wird Ihnen eine Reihe von Fragen präsentiert. Jede Frage hat vier mögliche Antworten, von denen Sie die richtige auswählen sollen. Jede Frage hat nur eine richtige Antwort.

Bitte verwenden Sie **keine Hilfsmittel** zur Beantwortung der Fragen, auch wenn Sie die Fragen nicht beantworten können.

Die Fragen gehören zu Aufgaben des Leseverständnisses. Da Sie jedoch keinen Zugang zu den Texten haben werden, auf die sich die Fragen beziehen, werden die meisten Fragen n**icht eindeutig beantwortbar** sein. Versuchen Sie dennoch, die Fragen so gut wie möglich zu beantworten, auch wenn Sie die Texte vorher nicht lesen können. Wenn Sie die Antwort nicht wissen, wählen Sie die Antwort, die Ihnen am wahrscheinlichsten erscheint.

Am Ende des Fragebogens haben Sie die Möglichkeit, einen Gutschein zu erhalten für die Beantwortung der Fragen. Drücken Sie “Weiter”, um fortzufahren.

## Appendix F Priors for Bayesian models

Table 25 presents the priors that have been used for the analysis in Section “Analyses of the data”.

**Table 25:** Overview of model priors

Log-linear regression	Logistic regression
$\beta _0\sim \mathcal {N}(6, 1.5)$	$\beta _0\sim \mathcal {N}(0, 4)$
$$\sigma \sim \mathcal {N}(0, 1)$$	
$$\beta _1\dots \beta _{11} \sim \mathcal {N}(0,1)$$	
$$\Omega \sim \textrm{LKJ}(1)$$	

$\mathcal {N}$ denotes the normal distribution parameterized with mean and standard deviation

$\beta _0$ denotes the global intercept, $\beta _1\dots \beta _{11}$ refer to the predictor coefficients described in Section “Reading time analysis”. $\sigma$ denotes the modeled variable’s standard deviation. $\Omega$ denotes the correlation matrix between the predictors

## Appendix G Attention checks for online survey

In order to validate the text comprehension questions, an online survey was conducted (Section “Validation of the text comprehension questions”). Attention check questions were added after six text questions. Those questions and answer options are listed below:

**Table:**

Was gibt 5 + 4:	Welches Wort ergibt “ein”
7	rückwärts gelesen?
9	nie
20	das
0	ich
	einen
Welches ist die Hauptstadt von Italien?	
Rom	Was ist das Gegenteil von
Neapel	“groß”?
Bologna	klein
Como	alt
	interessant
	laut
Welches ist eine Frucht?	Welches Tier hat Fell?
Apfel	Schlange
Fisch	Hund
Gurke	Blauwal
Weizen	Regenwurm

## Appendix H Down-sampling analysis

In this section, we present the results of the down-sampling analysis. This analysis follows the same procedure as the one presented in Section “Analyses of the data” except that we down-sampled the 16 oldest and four youngest participants in order to reduce the average difference between graduate and undergraduate students (graduates are typically older). Note that we removed four times as many older readers in order to obtain a more balanced set of graduate and undergraduate students. We present the results in Fig. 6. We see that the down-sampling did not affect the degree to which reading times are affected by expert reading, corroborating our finding that experts, regardless of age, make fewer first-pass regressions and exhibit shorter fixation times on texts in their discipline.

## Footnotes

1 https://pymovements.readthedocs.io/en/stable/

2 Dutch, English, Greek, German, Hebrew, Italian, Russian, Spanish, Turkish, Norwegian, Estonian and Finnish

3 Japanese, Arabic, Spanish, Portuguese, and Chinese

4 https://huggingface.co/benjamin/gerpt2-large

5 https://huggingface.co/benjamin/gerpt2

6 https://huggingface.co/LeoLM/leo-hessianai-7b

7 https://huggingface.co/LeoLM/leo-hessianai-13b

8 https://huggingface.co/bert-base-german-cased

9 https://spacy.io/

10 Data for 76 participants was collected, however, for one participant only the data for five out of 12 trials was available which is why this participant’s data was eventually excluded.

11 For some textual features like the reader discipline or the text domain, a numerical encoding was added for easy computational processing while still keeping an explicitly understandable text-encoded label (e.g., level of studies *physics*). As both features encode the same information, it won’t be explicitly stated in the following overview but is defined in the data repository.

12 download_data_files.py, please consult the README files in the PoTeC repository for further instructions.

13 Please note that the licenses per definition are for our own use only. That is: should you intend to publish any of the materials in another format somewhere else, you are responsible for clarifying the copyright situation.

14 https://www.cl.uzh.ch/en/research-groups/digital-linguistics/resources/potec.html

15 https://github.com/DiLi-Lab/PoTeC

16 https://pymovements.readthedocs.io/en/stable/reference/index.html

17 A detailed tutorial on how to use pymovements to download datasets in general can be found in the pymovements documentation: https://pymovements.readthedocs.io/en/stable/tutorials/R-tutorial.html and https://pymovements.readthedocs.io/en/stable/tutorials/public-datasets.html.

18 Note that in the data, there are two types of technical terms. Please refer to Section “Manual word-level annotation” and Table 4 for an explanation of those terms and all the features used in this present section.

19 Unknown words with lemma frequency 0 were assigned a pseudo-count of 1, which also corresponds to the lowest value $\ne 0$ encountered in the data set.

20 We used sentence-based surprisal values extracted from GPT-2 large.

21 https://pymovements.readthedocs.io/en/stable/reference/index.html

## Code Availability

All code is available in a GitHub repository at https://github.com/DiLi-Lab/PoTeC.
